# Effect of yoga on glycemic control in adults with type 2 diabetes mellitus: a Bayesian three-level meta-analysis of randomized controlled trials

**DOI:** 10.3389/fendo.2026.1889124

**Published:** 2026-08-11

**Authors:** Yixin Li, LongYu Nie, Ming Ang Li

**Affiliations:** 1 School of Sports Science, Qufu Normal University, Qufu, China; 2 College of Physical Education and Sports, Beijing Normal University, Beijing, China; 3 School of Physical Education, Wuhan Donghu University, Wuhan, China

**Keywords:** Bayesian meta-analysis, dose–response, fasting plasma glucose, glycated hemoglobin, physical activity, type 2 diabetes mellitus, yoga

## Abstract

**Background:**

Type 2 diabetes mellitus (T2DM) imposes a growing global burden, and glycemic control remains central to the prevention of long-term microvascular and cardiovascular complications. Yoga has been proposed as an adjunctive mind-body intervention, but existing meta-analytic evidence has been constrained by pooling approaches that ignore within-study dependency, dichotomized dose comparisons, and limited reporting of prediction intervals.

**Methods:**

PubMed, Embase, Web of Science, and the Cochrane Central Register of Controlled Trials were systematically searched through May 2026 for randomized controlled trials (RCTs) comparing structured yoga interventions with usual care or waiting-list control in adults with T2DM. The primary outcomes were glycated hemoglobin (HbA1c) and fasting plasma glucose (FBG). A Bayesian three-level random-effects model was fitted to accommodate within-study dependency. Categorical subgroup analyses, spline-based meta-regression, sensitivity analyses, publication bias assessments, and GRADE certainty ratings were also performed.

**Results:**

Twenty-eight RCTs comprising 2, 241 adults were included. Yoga reduced HbA1c by 0.64% (95% credible interval [CrI] −0.87 to −0.41) and FBG by 1.36 mmol/L (95% CrI −1.75 to −1.00), with direction probabilities of 100% and extreme Bayes factor evidence against the null hypothesis. The 95% prediction intervals remained below zero for both outcomes, although the upper bound for FBG approached the null. No clear evidence of effect modification was found by country, supervision modality, or the WHO threshold of 600 MET-min per week, although these subgroup comparisons were underpowered and evidence from outside India was limited. GRADE certainty was low for both HbA1c and FBG.

**Conclusions:**

In adults with T2DM, yoga was associated with clinically meaningful reductions in HbA1c and fasting glucose, with the largest benefits in patients with poorer baseline control, although the certainty of this evidence was low. These findings support consideration of yoga as a feasible adjunct to standard diabetes care rather than a fixed prescription.

**Systematic review registration:**

https://www.crd.york.ac.uk/PROSPERO/view/CRD420261391309, identifier CRD420261391309.

## 1 Introduction

Type 2 diabetes mellitus (T2DM) has become one of the most pressing public health challenges of this century. According to the 11th edition of the International Diabetes Federation Diabetes Atlas, an estimated 589 million adults aged 20–79 years were living with diabetes worldwide in 2024, and this number is projected to reach 853 million by 2050. China and India have long ranked among the countries with the highest absolute prevalence, and the Asia-Pacific region is expected to experience the steepest growth in diabetes burden over the next 25 years (1). Glycemic control remains central to the prevention of long-term microvascular and cardiovascular complications, with each 1% reduction in glycated hemoglobin (HbA1c) associated with a 21% decrease in the risk of any diabetes-related endpoint and a 37% decrease in microvascular complications (2). Long-term adherence to oral hypoglycemic agents nevertheless remains suboptimal (3), and contemporary antidiabetic drugs carry class-specific risks related to hypoglycemia, weight change, gastrointestinal tolerability, and cardiorenal safety. Even optimized pharmacological regimens differ substantially in their generalizability across patient populations (4). Accordingly, the American Diabetes Association, the World Health Organization, and the American College of Sports Medicine consistently recommend regular physical activity, alongside pharmacotherapy and nutritional intervention, as a core component of lifestyle management in T2DM (5–7).

Among the various modalities of physical activity, yoga has attracted sustained interest in endocrinology and rehabilitation medicine. Originating in ancient India, this mind-body practice integrates postures (asanas), breathing techniques (pranayama), and meditation. In contrast to conventional aerobic or resistance training, yoga combines muscular activity, autonomic regulation, and psychological relaxation (8), and may therefore influence glycemic control in patients with T2DM through multiple physiological pathways. In addition, yoga requires minimal equipment and space, imposes relatively low joint loading on older or obese patients, and is widely accepted across cultures, all of which support its feasibility in routine clinical practice.

Previous systematic reviews and meta-analyses of yoga in T2DM have provided initial support for this rationale (9–12), with reported reductions in HbA1c typically ranging from 0.4% to 0.7%. Several methodological limitations, however, persist across the existing evidence base. First, most reviews have relied on frequentist two-level random-effects models that do not adequately account for dependency among multiple arms or repeated time points within the same study, potentially underestimating between-study heterogeneity and overstating the precision of pooled estimates (13). Second, dose–response relationships have typically been examined through dichotomous subgroup comparisons, with nonlinear modeling rarely applied. Curvilinear features of the response profile are therefore difficult to detect (12, 14). Third, systematic appraisal of baseline heterogeneity, subgroup balance, and certainty of evidence remains incomplete, and few studies report prediction intervals, which limits the ability of clinicians to judge how reported effects may generalize to future patients and settings (15). Taken together, these limitations leave two clinically important questions unresolved. It remains unclear which patients benefit most from yoga, and at what dose.

To address these gaps, we conducted a systematic review and Bayesian three-level meta-analysis of yoga as an intervention for glycemic control in T2DM. The analysis was designed to address three linked questions. First, we estimated the pooled effect of yoga, relative to usual care, on HbA1c and fasting plasma glucose, together with the generalizability of that effect. Second, we examined whether intervention dose and baseline glycemic status function as clinically meaningful effect modifiers. Third, we assessed whether the overall certainty of evidence is sufficient to support the inclusion of yoga in clinical recommendation pathways for comprehensive T2DM management. The findings are intended to provide an actionable evidence base for endocrinologists, exercise specialists, and public health decision-makers.

## 2 Methods

### 2.1 Study protocol and reporting standards

This systematic review and meta-analysis was designed, conducted, and reported in accordance with the Preferred Reporting Items for Systematic Reviews and Meta-Analyses (PRISMA) 2020 statement, with analyses and reporting further informed by the Cochrane Handbook for Systematic Reviews of Interventions, version 6.4 (16, 17). The protocol was prospectively registered in the PROSPERO International Prospective Register of Systematic Reviews (registration number CRD 420261391309).

### 2.2 Search strategy

Two reviewers independently performed systematic searches of four electronic databases: PubMed, Embase, Web of Science Core Collection, and the Cochrane Central Register of Controlled Trials (CENTRAL). The search was completed in May 2026, with no restrictions on language or publication date. Search terms were organized into three conceptual blocks. For the population block, “type 2 diabetes mellitus” served as the core Medical Subject Headings (MeSH) term, supplemented by corresponding Emtree headings and free-text synonyms. For the intervention block, “yoga” was the core controlled vocabulary term, expanded with free-text variants covering individual yoga styles and specific practice components. For the study design block, controlled vocabulary terms and free-text terms relating to randomized controlled trials were combined. Each database was searched using both controlled vocabulary and free-text strategies (MeSH for PubMed, Emtree for Embase), and the search syntax was tailored to the conventions of each database (full strategies are provided in **Supplementary File 1**). Reference lists of the included studies and relevant reviews were also screened manually to identify potentially eligible studies missed by the electronic searches.

### 2.3 Eligibility criteria

Eligibility criteria were specified according to the PICOS framework.

Population (P). Adults aged 18 years or older with a clinical diagnosis of T2DM were eligible, with no restriction on sex, ethnicity, geographical region, or disease duration.

Intervention (I). The intervention arm was required to receive a structured yoga-based program incorporating postures (asanas), breathing techniques (pranayama), or meditation, applied either as a single component or as a composite practice. Yoga had to be delivered as a standalone active intervention, or as an independently extractable yoga arm in multi-arm trials. Studies in which yoga was inseparably combined with another active therapy were excluded.

Comparator (C). The comparator included usual care, health education, a waiting list, or a nonexercise control. Yoga had to be the only planned difference between groups. Background pharmacotherapy and medication changes were assessed separately because reporting was often incomplete.

Outcomes (O). The primary outcomes were HbA1c and fasting plasma glucose (FBG). At least one of these outcomes had to be reported, with pre- and post-intervention means, standard deviations, and sample sizes either available directly or recoverable from the data presented.

Study design (S). Eligibility was restricted to randomized controlled trials (RCTs).

Studies were excluded for any of the following reasons. They enrolled populations other than T2DM. Yoga was combined with another active therapy in a manner that prevented isolation of its independent effect. They used non-randomized designs such as single-arm studies or narrative reviews. Data could not be extracted and remained unavailable after contact with the authors. Finally, when studies represented duplicate publications, the most informative version was retained.

### 2.4 Study selection and data extraction

**Figure 1:**
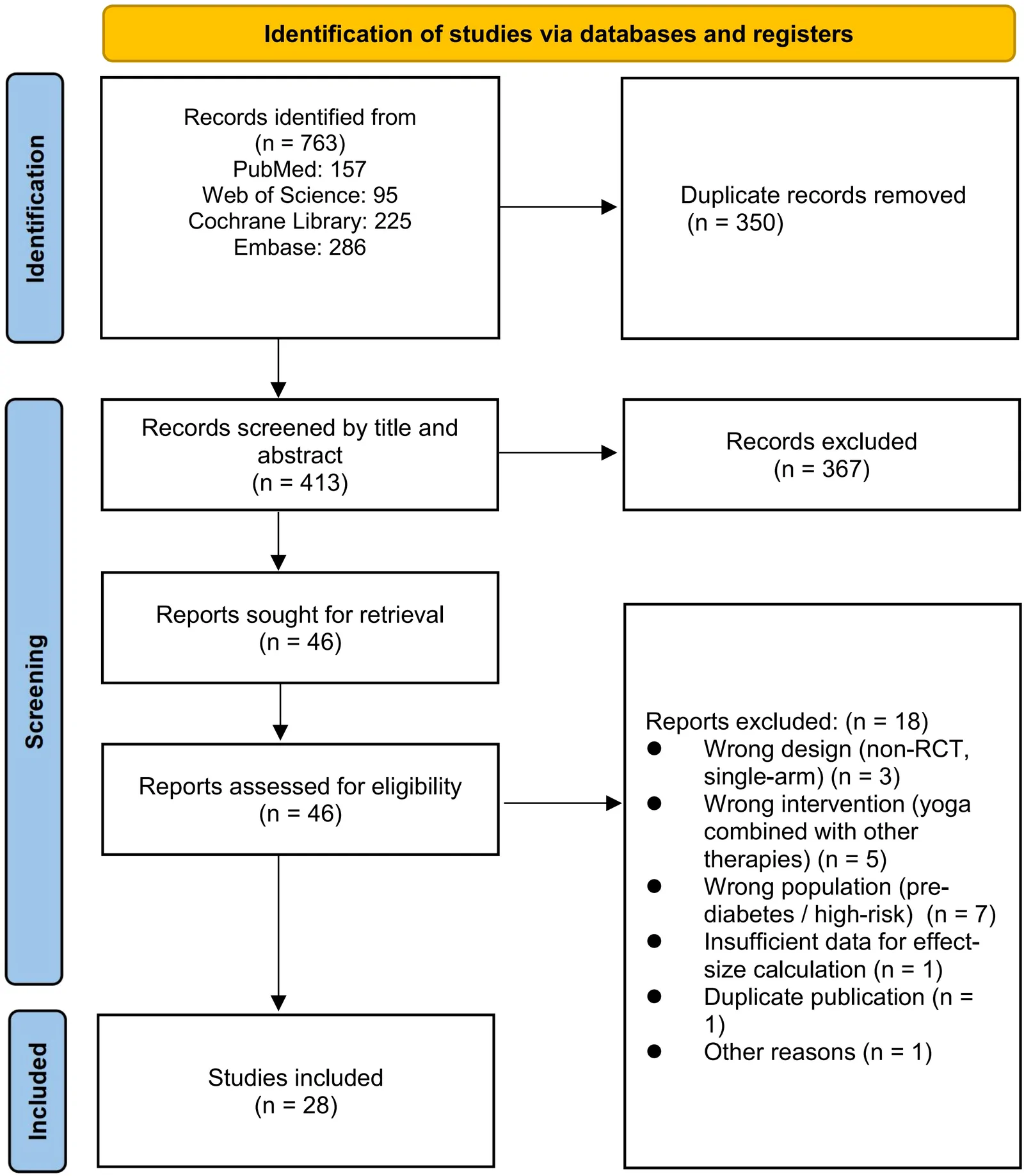
PRISMA 2020 flow diagram. The systematic search of four electronic databases (PubMed, Embase, Web of Science, and Cochrane Library) yielded 763 records. After removal of 350 duplicates, 413 records entered title and abstract screening, and 367 were excluded. Forty-six studies underwent full-text review, of which 18 were excluded for failing to meet eligibility criteria (reasons shown in the corresponding branches). Twenty-eight randomized controlled trials were ultimately included in the quantitative synthesis.

All retrieved records were imported into EndNote for deduplication. Two reviewers then independently screened titles and abstracts, after which potentially eligible studies underwent full-text review. Disagreements between the two reviewers were resolved by consultation with a third experienced reviewer. The complete selection process is summarized in the PRISMA flow diagram (**Figure 1**). Data extraction was performed independently by two reviewers using a piloted standardized form, capturing five categories of information: (1) study characteristics, including first author, year of publication, country or region, study design, sample size, and follow-up duration; (2) demographic and baseline characteristics, including mean age, proportion of female participants, mean body mass index (BMI), and baseline HbA1c and FBG; (3) intervention characteristics, including yoga style, duration of each session, weekly frequency, total intervention duration in weeks, and whether sessions were supervised; (4) outcome data, including pre- and post-intervention means, standard deviations, and sample sizes for HbA1c and FBG; (5) background glucose-lowering therapy, medication stability requirements, dose adjustment policies, and reported between-group differences in medication changes. Extracted data were cross-checked between the two reviewers, with discrepancies resolved by a third reviewer.

### 2.5 Effect size measurement and dose coding

The mean difference (MD) was used as the effect size, retaining the original measurement scales. HbA1c was expressed in percentage points and FBG in mmol/L, with negative values indicating improvement in the yoga group relative to the control group. When HbA1c was reported in mmol/mol, values were converted to National Glycohemoglobin Standardization Program (NGSP) percentage units using the formula NGSP% = (mmol/mol × 0.09148) + 2.15 (18). FBG values reported in mg/dL were converted to mmol/L by multiplying by 0.0555, equivalent to division by 18.018. For studies reporting only standard errors, 95% confidence intervals, or interquartile ranges, standard deviations were back-calculated using the methods recommended in the Cochrane Handbook. The standard deviation of the pre- to post-intervention change score was derived using the standard formula incorporating the baseline and post-intervention standard deviations and the assumed pre–post correlation coefficient (19). Because within-participant correlations were not reported, a moderate correlation of 0.5 was used as the primary assumption. Sensitivity analyses tested values of 0.3, 0.7, and 0.9 (see Section 2.7). Following diabetes care guidance and the effect-size ranges reported in previous exercise-based meta-analyses, reductions of ≥0.5% in HbA1c and ≥1.0 mmol/L in FBG were treated as clinically interpretable reference thresholds when interpreting the results (20, 21).

To standardize the description of yoga dose across studies, metabolic equivalent (MET) values were assigned to each yoga protocol according to the 2024 Adult Compendium of Physical Activities (22). Predominantly posture-based general or Hatha yoga protocols were coded as 2.5 METs, breathing-focused pranayama protocols as 1.5 METs, meditation- or gazing-based practices as 1.3 METs, low-intensity seated or restorative practices as 2.0 METs, and Surya Namaskar or flow-based practices as 3.3 METs when explicitly described. Where study descriptions supported a specific value, that value was used. Otherwise, the default value of 2.5 METs for unspecified or mixed yoga was applied. The weekly dose was then computed as MET × session duration (min) × weekly frequency, expressed in MET-min/week. Studies were classified into higher- and lower-dose subgroups using the World Health Organization (WHO) 2020 physical activity guideline threshold of 600 MET-min/week.

### 2.6 Risk of bias assessment

Risk of bias in the included studies was assessed using the Cochrane Risk of Bias 2.0 (RoB 2) tool (23), with the results visualized and summarized using the robvis package (24). Five domains were appraised: the randomization process, deviations from intended interventions, missing outcome data, measurement of the outcome, and selection of the reported result. An overall judgement of “low risk”, “some concerns”, or “high risk” was assigned to each study. In accordance with Cochrane Handbook guidance, risk of bias was evaluated separately for each outcome, and the results for HbA1c and FBG are reported in **Supplementary Files 3**, **4**, respectively. Two reviewers performed the assessments independently, with discrepancies resolved through consultation with a third experienced reviewer.

### 2.7 Statistical analysis

A Bayesian three-level random-effects meta-analytic model was used to accommodate the hierarchical dependency arising from multiple arms or repeated time points within the same study (25). The fixed-effects component included group (yoga vs. control) as the predictor of interest. The random-effects component used nested levels for study and effect size, with the former capturing between-study heterogeneity (τ_study) and the latter capturing within-study clustering across arms or time points (τ_within) (26). Observation-level variance was fixed at the sampling variance derived from the reported standard error for each effect estimate. Weakly informative priors were specified for both outcomes. In the HbA1c model, the intercept and regression coefficients followed Normal(0, 1), and the heterogeneity standard deviations followed zero-truncated Cauchy(0, 0.5) priors. For FBG, the corresponding prior scales were doubled to Normal(0, 2) and zero-truncated Cauchy(0, 1) to accommodate the larger natural variation of glucose values on the mmol/L scale, consistent with recommendations for weakly informative priors on heterogeneity parameters in Bayesian random-effects meta-analysis (27). Models were fitted with the brms package using the Hamiltonian Monte Carlo No-U-Turn Sampler (NUTS) implemented in Stan, with four chains of 3, 000 iterations each (the first 1, 500 iterations serving as warm-up) and adapt_delta set to 0.99 (28–30). Convergence was evaluated using rank-normalized $\hat{R}$ values below 1.01, together with bulk and tail effective sample size diagnostics. All monitored parameters had effective sample sizes exceeding the commonly recommended minimum of 400 (31). The primary analysis reported the pooled mean difference together with its 95% credible interval, 95% prediction interval, direction probability, Bayes factor (BF_10_), Bayesian R², and both heterogeneity standard deviations. Direction probabilities and credible intervals were computed with the bayestestR package (32). Parameter-level Bayes factors were derived from the Savage–Dickey density ratio in the bayestestR package, whereas model-level Bayes factors based on marginal likelihoods were computed with the bridgesampling package (32, 33).

Subgroup analyses were performed for clinically relevant categorical moderators, including study country, supervision status, and a weekly-dose threshold of 600 MET-min/week, corresponding to the lower bound of the WHO 2020 recommendation for moderate-intensity-equivalent physical activity (5), overall risk-of-bias rating, and yoga style. To avoid unreliable conclusions from severely imbalanced comparisons, a prespecified balance criterion was applied. Each level was required to contain at least four studies, and the smallest level was required to account for at least 20% of the included studies. For each moderator meeting these criteria, a model incorporating a group-by-moderator interaction term was fitted, and the Bayes factor for the interaction model versus the no-interaction model was computed via bridge sampling of the marginal likelihood. BF_10_ < 1 was interpreted as evidence favoring the no-interaction model over the interaction model (33, 34). Meta-regression was conducted for seven continuous moderators: baseline outcome value, weekly session frequency, session duration, intervention duration in weeks, mean age, proportion of female participants, and mean BMI. All continuous variables were z-standardized before fitting, so that each β coefficient represents the change in effect per one standard deviation increase (35). Each variable was fitted under three functional forms: linear, thin-plate spline with k = 3, and thin-plate spline with k = 4. The spline forms were fitted only when the variable had six or more unique values. Model selection used the leave-one-out cross-validation information criterion (LOO-IC) implemented in the loo package, with the model showing the lowest LOO-IC retained (36).

Publication bias was assessed using study-level effect sizes, defined as the between-group difference in change scores, with corresponding standard errors derived by pooling the standard errors of the two arms. These analyses were performed with the metafor package (37). Contour-enhanced funnel plots with 95% and 99% pseudo-confidence bands were generated, and Egger’s regression and Begg’s rank correlation tests were applied, with p < 0.05 considered indicative of funnel asymmetry (38–40). To assess small study effects and potentially missing studies, we conducted trim and fill analyses using the L0 and R0 estimators and performed PET PEESE analysis. PEESE was used when the PET intercept was significant, and PET was used otherwise. These analyses used study level between group differences in pre to post change scores. Three sensitivity analyses were performed. The first sequentially excluded each study and refitted the primary model. The second recalculated change score standard deviations using pre-post correlations of 0.3, 0.7, and 0.9. The third refitted the model after excluding studies at high overall risk of bias for each outcome. Finally, the certainty of evidence for each outcome was rated using the Grading of Recommendations Assessment, Development and Evaluation (GRADE) framework (41), with randomized evidence initially rated as high certainty. Downgrading was considered for risk of bias, inconsistency, indirectness, imprecision, and publication bias. Risk of bias was judged by the nature and likely impact of identified limitations on the pooled estimate. Sensitivity analyses excluding high risk studies assessed their influence on the results. Inconsistency was judged primarily by whether the prediction interval crossed the null. All analyses were performed in R version 4.5.3.

## 3 Results

### 3.1 Search results

The systematic search across the four databases yielded 763 records (PubMed, 157; Embase, 286; Web of Science, 95; Cochrane Library, 225). After removal of 350 duplicates, 413 records entered title and abstract screening, of which 367 were excluded according to the prespecified criteria, leaving 46 studies for full-text review. Eighteen studies were subsequently excluded for the following reasons: non-T2DM populations (n = 7), yoga combined with another active therapy (n = 5), non-randomized design (n = 3), data not extractable (n = 1), duplicate publication (n = 1), and other reasons (n = 1). Twenty-eight RCTs were ultimately included in the quantitative synthesis. The full selection process is illustrated in the PRISMA flow diagram (**Figure 1**).

### 3.2 Characteristics of the included studies

The 28 RCTs comprised 2, 241 adults with T2DM (1, 123 in the yoga arms and 1, 118 in the control arms). Publication years ranged from 2008 to 2026, and the included studies were geographically concentrated in India (20 trials), with the remaining trials conducted in Cuba, the United Kingdom, the United States, Iran, Indonesia, Egypt, Japan, and Türkiye. Hatha yoga and composite yoga programs were the most commonly used styles. Dose parameters varied considerably across studies. Session duration ranged from 10 to 90 minutes (median 60 minutes), weekly frequency from 0.5 to 7 sessions (median 3 sessions per week), and total intervention duration from 4 to 24 weeks (median 12 weeks). Twenty-two studies reported HbA1c, 24 studies reported FBG, and 18 studies reported both outcomes. Background pharmacotherapy was inconsistently reported. Six trials confirmed no medication changes in either group during follow up. Three required stable therapy at enrollment but did not report subsequent adjustments. Three omitted baseline glucose lowering regimens, and one reported inconsistent information. Most remaining trials reported only broad drug classes or continued standard care. Laimujam 2025 showed a marked baseline imbalance in metformin use, while Subramani 2025 reported more medication dose reductions in the yoga group. Detailed characteristics of the included studies are provided in **Supplementary File 2**.

### 3.3 Risk of bias

As specified in the Cochrane Handbook, risk of bias was appraised separately for the HbA1c and FBG outcomes, and the distributions across studies were similar for the two subsets. In the HbA1c subset (22 studies), 20 studies were rated as having “some concerns” and two studies (Mullur 2016 and Subramani 2025) were judged to carry a “high” overall risk. In the FBG subset (24 studies), 23 studies were rated as having “some concerns” and one study (Mullur 2016) was judged “high”. No study reached a “low” overall rating in either subset. The main concerns involved incomplete reporting of randomization, deviations from intended interventions, missing data, and selective reporting. Outcome measurement was at low risk because HbA1c and FBG were assessed using objective laboratory methods. Detailed appraisal tables are provided in **Supplementary File 3**, and the summary plots in **Supplementary File 4**.

### 3.4 Main analyses

In the HbA1c subset (22 studies, 44 arm-level observations), yoga produced a credible reduction in HbA1c, with a pooled MD of −0.64% (95% credible interval [CrI] −0.87 to −0.41), a direction probability (pd) of 100%, a parameter-level BF_10_ of approximately 2.1 × 10³, and a model-level BF_10_ of approximately 1.2 × 10^4^, indicating extreme evidence against the null hypothesis. The 95% prediction interval was [−1.17, −0.09] and lay entirely below zero. Between-study heterogeneity (τ_study) was 0.21 and within-study heterogeneity (τ_within) was 0.32, with a Bayesian R² of 0.83 (95% CrI 0.73–0.92). All $\hat{R}$ values were below 1.01. The magnitude of this reduction exceeded the 0.5% threshold commonly regarded as clinically meaningful.

In the FBG subset (24 studies, 48 arm-level observations), yoga similarly produced a credible reduction in fasting plasma glucose, with a pooled MD of −1.36 mmol/L (95% CrI −1.75 to −1.00), a pd of 100%, a parameter-level BF_10_ of approximately 5.4 × 10^5^, and a model-level BF_10_ of approximately 4.2 × 10^5^. The 95% prediction interval was [−2.72, −0.03] and lay entirely below zero. Heterogeneity was higher than that observed in the HbA1c model, with τ_study = 0.62 and τ_within = 0.54. The Bayesian R² was 0.74 (95% CrI 0.59–0.88), and all $\hat{R}$ values were below 1.01. The magnitude of the reduction exceeded the prespecified clinically interpretable reference threshold of 1.0 mmol/L.

**Figure 2:**
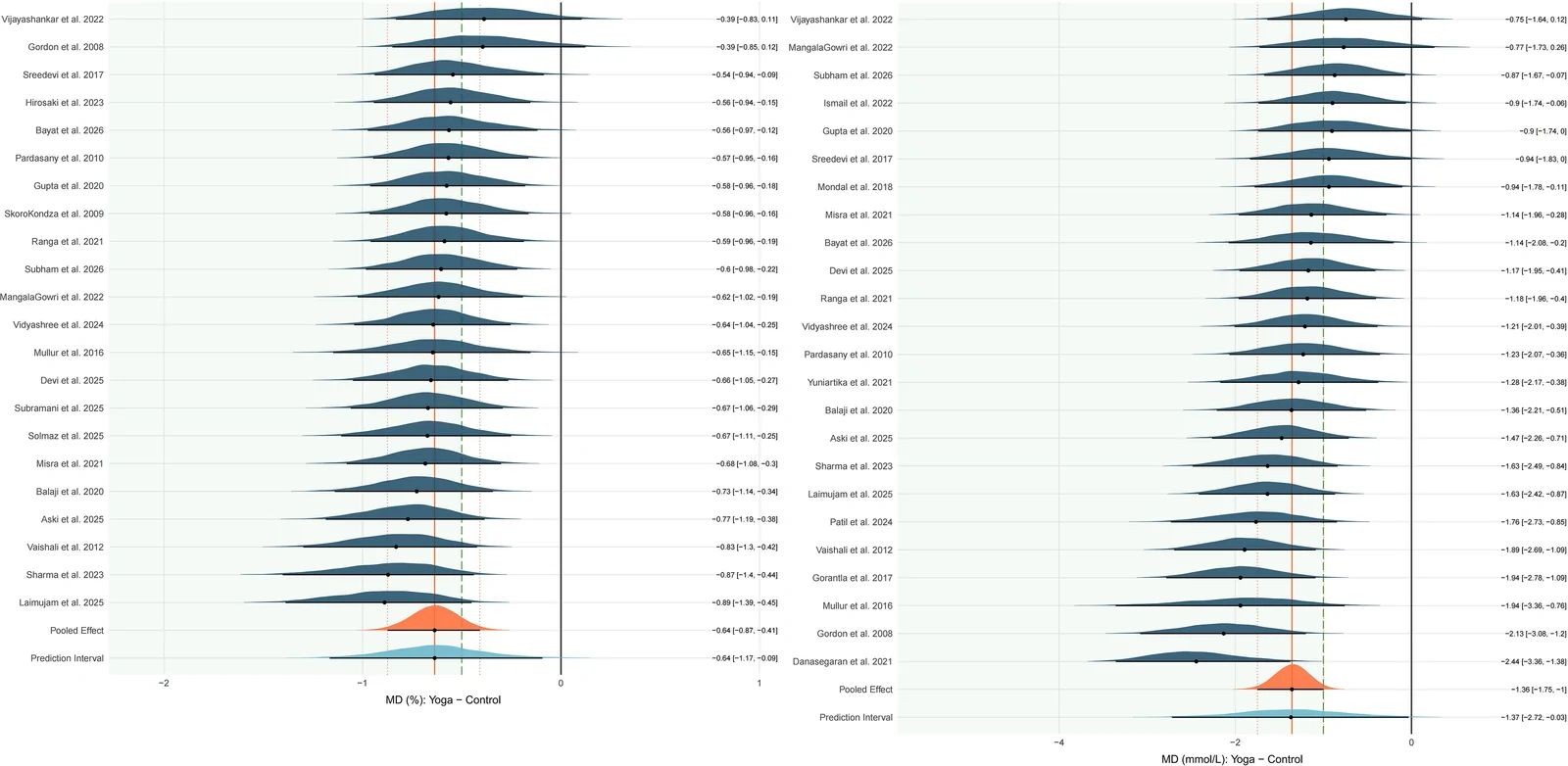
Forest plots of the effect of yoga on HbA1c and fasting blood glucose (FBG). **(A)** Pooled effect in the HbA1c subset; **(B)** pooled effect in the FBG subset. Each study row displays the posterior distribution of the study-specific mean difference (MD), with the point estimate and 95% credible interval (CrI) shown alongside the density curve. The orange density curve represents the posterior distribution of the pooled effect, and the light blue density curve represents the predictive distribution for a future study. Its horizontal span shows the 95% prediction interval. Vertical reference lines indicate the pooled estimate, the bounds of the pooled 95% CrI, and the null reference. Negative values indicate greater improvement in the yoga arm than in the control arm.

Forest plots for both outcomes are presented in **Figure 2**. Detailed model diagnostics and posterior density plots are provided in **Supplementary Files 5**, **6**, and the numerical summary of the main analyses is given in **Table 1**.

**Table 1:** Main analysis results and GRADE certainty of evidence.

Outcome	k (studies)	Arms	Pooled MD [95% CrI]	95% PI	τ_study	τ_within	pd	BF_10_ (parameter)	BF_10_ (model)	Bayesian R²	GRADE
HbA1c (%)	22	44	−0.64 [−0.87, −0.41]	[−1.17, −0.09]	0.21	0.32	100%	2.1 × 10³	1.2 × 10^4^	0.83 [0.73, 0.92]	⊕⊕○○ Low
FBG (mmol/L)	24	48	−1.36 [−1.75, −1.00]	[−2.72, −0.03]	0.62	0.54	100%	5.4 × 10^5^	4.2 × 10^5^	0.74 [0.59, 0.88]	⊕⊕○○ Low

Effect size is the mean difference (MD) on the original measurement scale (HbA1c %, FBG mmol/L); negative values favour yoga. Model: three-level Bayesian random-effects meta-analysis [md | se(se_md) ~ 1 + group + (1 | studyID/es_id)], 4 chains × 3000 iterations (warmup 1500), all $\hat{R}$ < 1.01. τ_study, between-study heterogeneity SD; τ_within, within-study (multi-arm/multi-timepoint) SD. PI, 95% prediction interval for a future study; pd, probability of direction; BF_10_, Bayes factor against the null (Savage–Dickey density ratio for the parameter; bridge sampling for the model). GRADE certainty for HbA1c was downgraded one level for risk of bias and one level for suspected publication bias. Excluding the two high-risk studies produced a similar pooled estimate. Certainty for FBG was likewise downgraded one level for risk of bias and one level for suspected publication bias. No downgrading was applied for inconsistency because the 95% prediction interval remained below zero for both outcomes. A full evidence profile is provided in **Supplementary File 16**. ⊕⊕○○ Low.

### 3.5 Subgroup analyses

Among the five prespecified subgroup comparisons, three met the balance criterion, defined as at least four studies per level with the smallest level accounting for at least 20% of studies. These three were country, supervision modality, and the WHO weekly-dose threshold of 600 MET-min/week. The risk-of-bias and yoga-style comparisons were automatically skipped because of severe imbalance across levels.

Pooled effects in the Indian and non-Indian subgroups were highly consistent. In the HbA1c subset, the pooled MD was −0.61% for India (k = 16) and −0.66% for non-India (k = 6). Estimates were similar, but only six non-Indian studies contributed to the HbA1c analysis and five to the FBG analysis. The comparison was therefore underpowered and does not establish cross-cultural equivalence. The supervision comparison met the balance criterion only in the FBG subset. The fully supervised stratum (k = 18) yielded a pooled MD of −1.36 mmol/L, and the supervised-plus-home stratum (k = 6) a pooled MD of −1.36 mmol/L, with virtually overlapping effects. For the WHO dose threshold (≥ 600 vs. < 600 MET-min/week), the pooled MD in the HbA1c subset was −0.63% for studies above the threshold (k = 8) and −0.63% for studies below it (k = 14). In the FBG subset, the corresponding values were −1.50 mmol/L (k = 8) and −1.25 mmol/L (k = 16), again with overlapping credible intervals.

**Figure 3:**
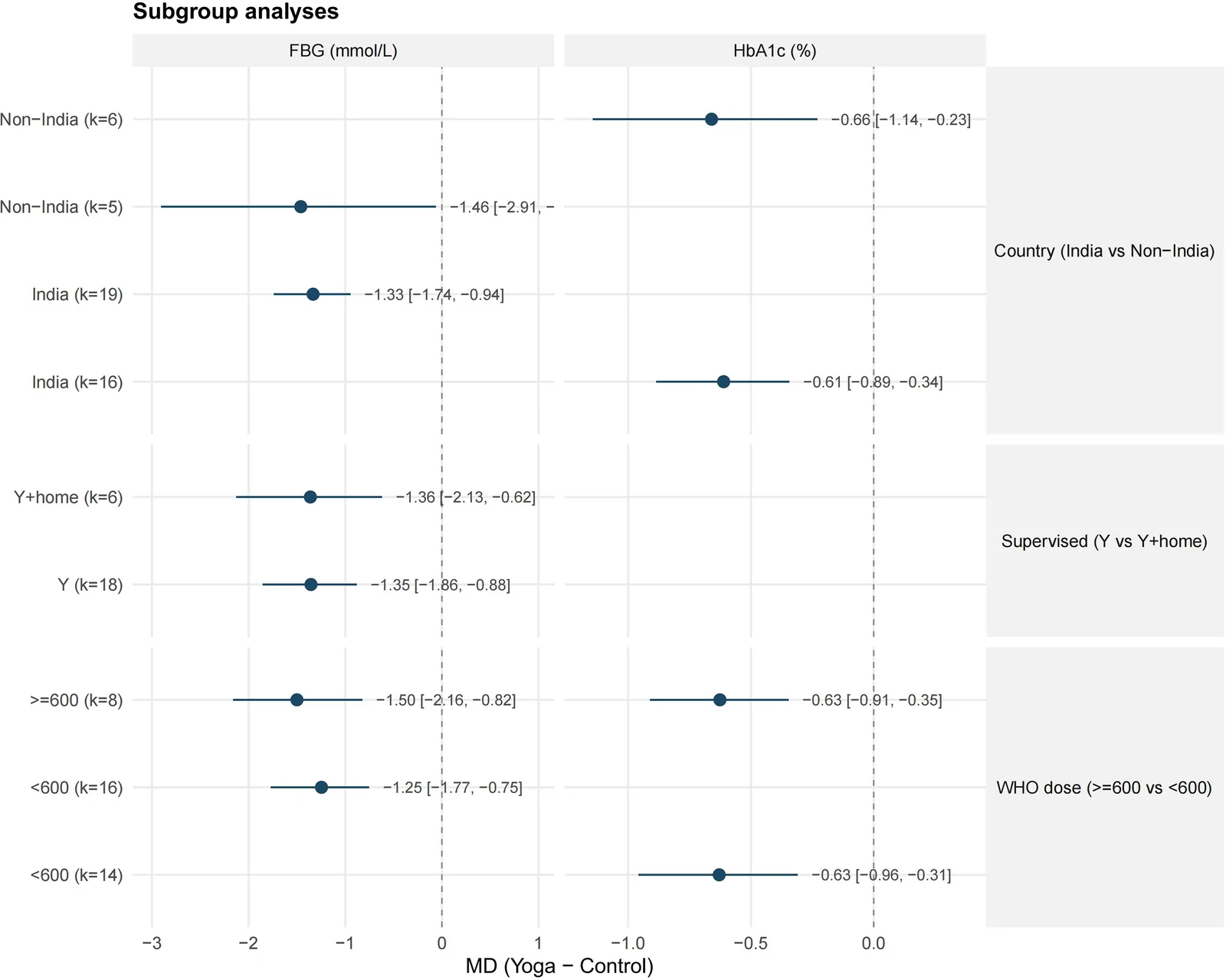
Subgroup forest plots for categorical moderators meeting the prespecified balance criterion. Columns correspond to the two outcomes, with fasting blood glucose (FBG) shown on the left and HbA1c shown on the right. The three facet blocks represent categorical moderators that satisfied the balance criterion, defined as at least four studies per level and the smallest level accounting for at least 20% of the included studies: country (India vs. non-India), supervision modality (fully supervised vs. supervised plus home practice), and the WHO weekly-dose threshold (≥600 vs. <600 MET-min/week). Supervision modality met the balance criterion only in the FBG subset. Points and horizontal lines denote the subgroup-pooled mean difference (MD) and its 95% credible interval (CrI), and k denotes the number of studies in each subgroup level. Across both outcomes, all subgroup interaction Bayes factors were below 1 (range 0.19–0.30), indicating evidence favoring the no-interaction model over the interaction model. Negative values favor yoga.

Interaction Bayes factors for all subgroups passing the balance criterion ranged from 0.19 to 0.30, with all BF_10_ values below 1. Because each of these comparisons rested on small strata, these values indicate an absence of evidence for effect modification rather than evidence of equivalence. The subgroup analysis results are presented in **Figure 3**.

### 3.6 Meta-regression

Meta-regression was performed for seven continuous moderators, with the functional form selected by minimum LOO-IC. Of the 14 analyses conducted (7 moderators across 2 outcomes), three yielded 95% credible intervals that excluded zero. These were baseline HbA1c in the HbA1c subset, together with baseline FBG and session duration in the FBG subset. The corresponding standardized β coefficients were −0.20, −0.49, and +0.95, respectively, with the thin-plate spline at k = 3 selected as the best-fitting form in each case.

**Figure 4:**
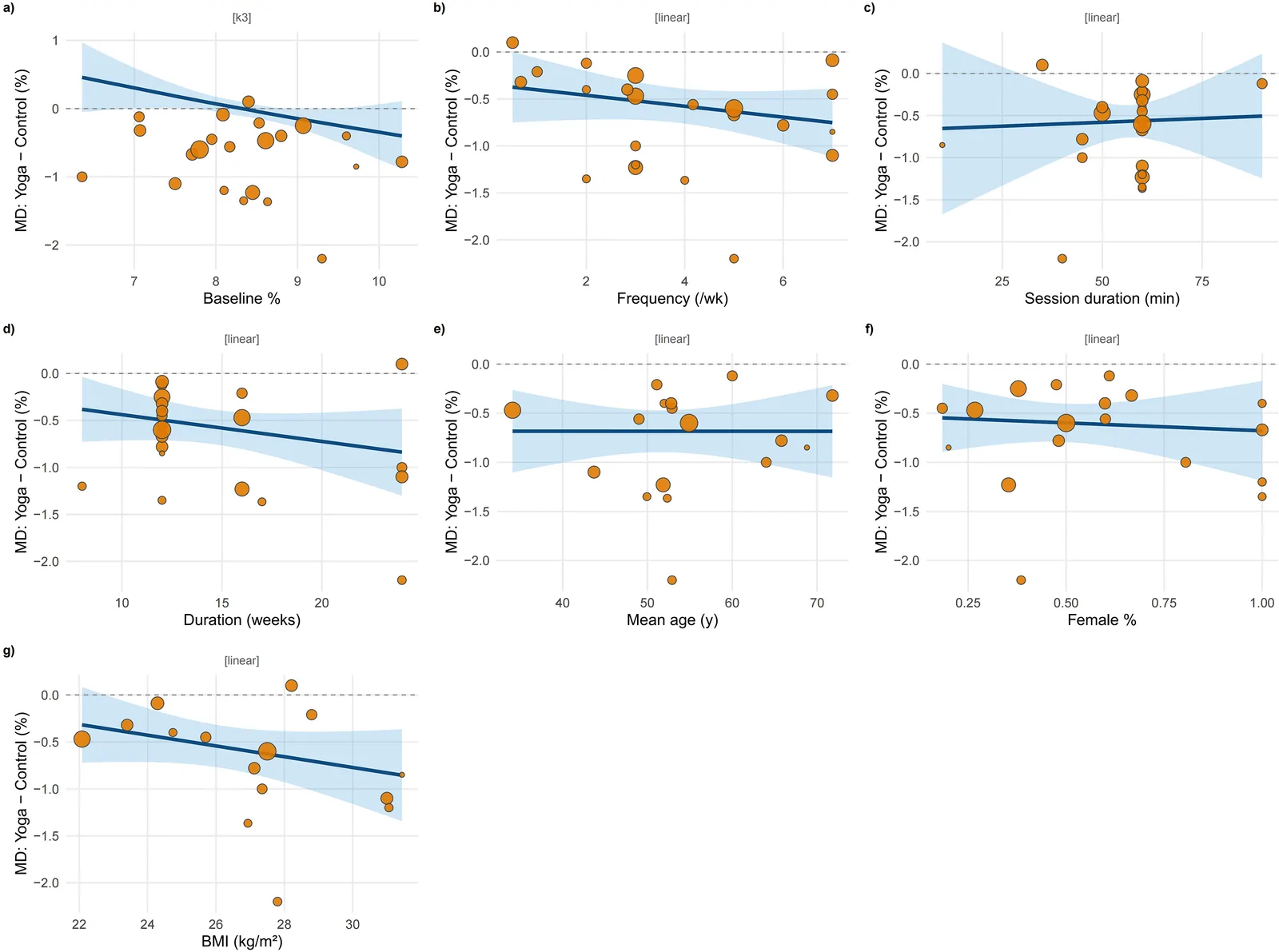
Conditional effect curves for the seven continuous moderators in the HbA1c subset. The seven panels show: **(a)** baseline HbA1c; **(b)** weekly session frequency; **(c)** session duration; **(d)** intervention duration in weeks; **(e)** mean age; **(f)** proportion of female participants; and **(g)** mean BMI. The bracketed label above each panel indicates the functional form selected by minimum LOO-IC: linear or thin-plate spline with k = 3. The dark-blue solid line represents the conditional predicted mean difference of yoga relative to control, and the light-blue shaded band represents the corresponding 95% credible interval (CrI). Orange points denote the between-arm “yoga minus control” difference for each study, with point size proportional to study precision (1/SE²). The dashed grey horizontal line denotes the null reference. Negative values indicate greater improvement in the yoga arm than in the control arm. Consistent with the meta-regression results, baseline HbA1c was identified as the only credible study-level moderator in the HbA1c subset.

**Figure 5:**
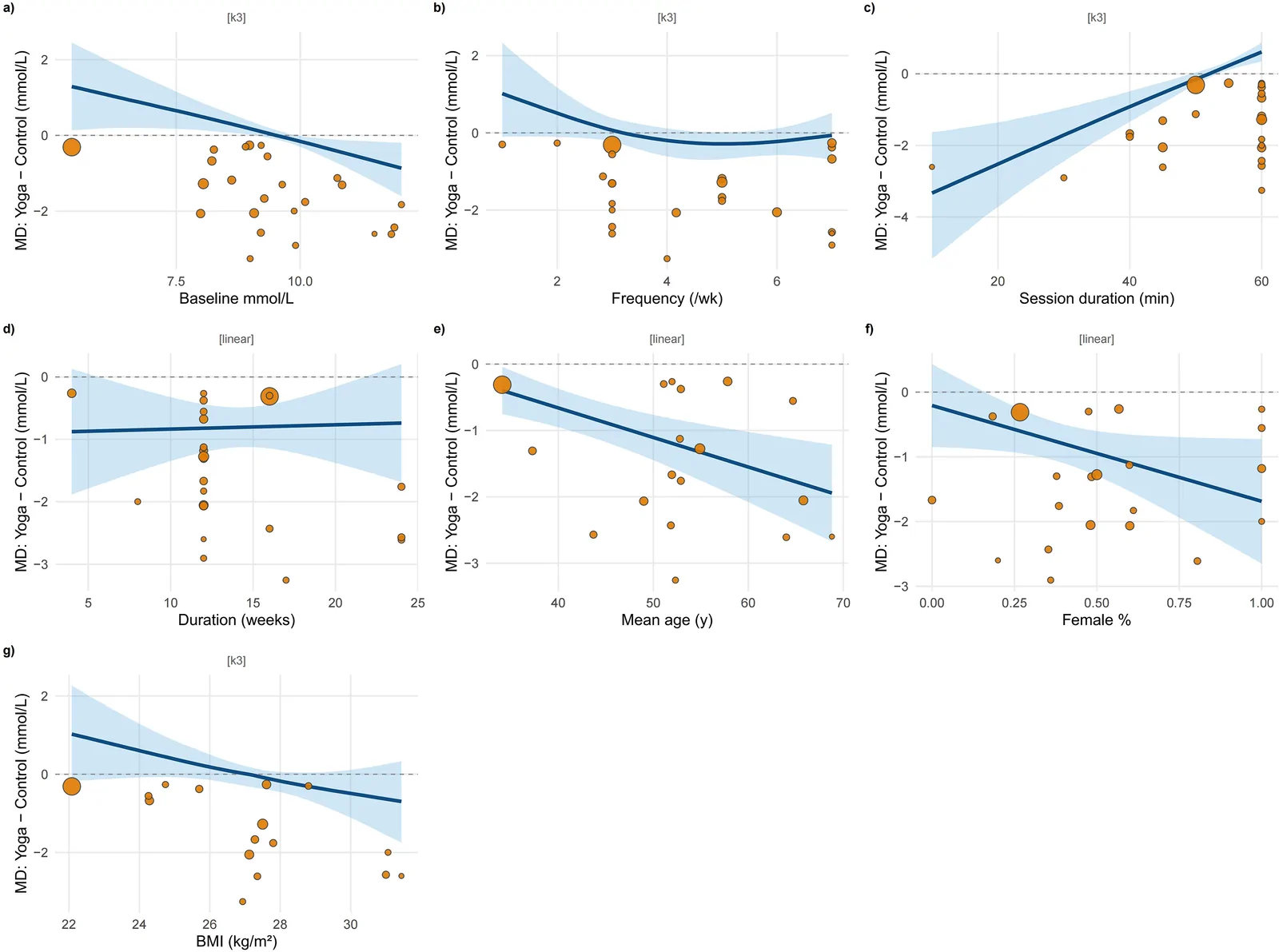
Conditional effect curves for the seven continuous moderators in the FBG subset. The seven panels show: **(a)** baseline FBG; **(b)** weekly session frequency; **(c)** session duration; **(d)** intervention duration in weeks; **(e)** mean age; **(f)** proportion of female participants; and **(g)** mean BMI. Layer encoding follows that of **Figure 4**. The bracketed label above each panel indicates the functional form selected by minimum LOO-IC: linear or thin-plate spline with k = 3. Baseline FBG and session duration were associated with treatment effects at the study level. However, the session duration analysis included only seven distinct values and may have been confounded by study size, risk of bias, adherence, and related intervention characteristics. It does not establish that shorter sessions are more effective or define an optimal duration. Negative values indicate greater improvement in the yoga arm than in the control arm.

In the HbA1c subset, baseline HbA1c was the only credible moderator. As baseline HbA1c increased from 7% to 10%, the predicted effect of yoga relative to control shifted monotonically from approximately +0.4% to approximately −0.5%, indicating progressively greater benefit at higher baseline values. Credible intervals for the remaining moderators (weekly frequency, session duration, intervention duration in weeks, mean age, proportion of female participants, and BMI) all included zero. In the FBG subset, baseline FBG and session duration emerged as credible moderators. As baseline FBG increased from 7.5 mmol/L to 11 mmol/L, the predicted reduction strengthened monotonically from approximately 0 to approximately 2.5 mmol/L. Session duration showed a nonlinear study-level association with effect size. Studies prescribing shorter sessions, especially 20 to 30 minutes, tended to report greater reductions in FBG. The predicted effect moved toward the null as session duration increased. However, this exploratory finding was based on only seven distinct duration values and does not indicate that shorter sessions are inherently more effective. Session duration was not randomized and may have been confounded by study size, risk of bias, adherence, baseline glycemia, weekly frequency, yoga intensity, program duration, and other study-level factors. The trial with the shortest sessions prescribed 10 minutes per session, included only 10 participants, and was the only FBG study rated at high overall risk of bias. In prespecified robustness checks, excluding this trial attenuated the session-duration coefficient from 0.95 to 0.67, although its 95% CrI remained above zero (0.34 to 1.01). Leave-one-out identified it as the single most influential study, and the association persisted after excluding all below-median-sized studies (0.74, 0.30 to 1.16) and after weighting by study precision (0.97, 0.58 to 1.36). Its direction was therefore robust, but its magnitude was partly driven by this one small high-risk trial (**Supplementary File 7**). This finding should therefore be considered hypothesis-generating and was not used to recommend an optimal session duration. Conditional effect curves for the HbA1c and FBG subsets are presented in **Figures 4** and **5**, respectively, with detailed numerical results in **Supplementary File 7**.

### 3.7 Publication bias

For HbA1c, the funnel plot was approximately symmetric. Neither Egger’s test (p = 0.253) nor Begg’s test (p = 0.197) indicated asymmetry. In secondary adjustment analyses, trim and fill imputed three studies with the L0 estimator and none with the R0 estimator. The adjusted estimates were −0.54% (95% CI −0.78 to −0.29) and −0.64% (95% CI −0.85 to −0.43), respectively. The PET PEESE conditional estimate was −0.32% (95% CI −0.73 to 0.10). Although neither asymmetry test was significant and trim-and-fill estimates remained below zero, this conditional estimate crossed the null, indicating that small-study effects could not be fully excluded for HbA1c. For FBG, Egger’s test indicated asymmetry (p < 0.001), whereas Begg’s test did not (p = 0.941), suggesting possible small study effects rather than confirmed publication bias. Trim and fill imputed four studies with the L0 estimator and five with the R0 estimator. The adjusted estimates were −1.24 mmol/L (95% CI −1.60 to −0.89) and −1.17 mmol/L (95% CI −1.54 to −0.81), respectively. The PET PEESE estimate was −1.27 mmol/L (95% CI −1.66 to −0.89). These estimates were smaller than the observed REML estimate of −1.38 mmol/L (95% CI −1.73 to −1.03), but all remained below zero. Small study effects may therefore have inflated the magnitude of the FBG effect without changing its direction. Full results are provided in **Supplementary File 15**.

### 3.8 Sensitivity analyses

In the leave one out analysis, pooled MDs ranged from −0.57% to −0.67% for HbA1c and from −1.30 to −1.43 mmol/L for FBG. Varying the assumed pre and post correlation from 0.3 to 0.9 changed the estimates by only 0.07% and 0.13 mmol/L, respectively. After excluding high risk studies, the pooled MD was −0.67% for HbA1c (95% CrI −0.91 to −0.45) and −1.34 mmol/L for FBG (95% CrI −1.73 to −0.97). All credible intervals remained below zero, and the primary findings were unchanged. Detailed results are provided in **Supplementary Files 10**–**14**.

### 3.9 Certainty of evidence

Certainty was rated separately for each outcome using a criterion-by-criterion approach. The full evidence profile with per-domain judgements is provided in **Supplementary File 16**.

For HbA1c, certainty was downgraded one level for risk of bias and one level for suspected publication bias, giving low certainty. No study reached low overall risk. Twenty of 22 studies carried some concerns and two were at high risk, arising mainly from incomplete reporting of randomization and of deviations and from the unavoidable lack of blinding in a mind-body intervention. Outcome measurement was at low risk in every study because HbA1c is an objective standardized laboratory measure. A two-level downgrade for risk of bias was considered but rejected, because only two studies were at high risk, their exclusion left the estimate essentially unchanged (−0.67%, 95% CrI −0.91 to −0.45), and no single domain reached high risk across the majority of studies. Inconsistency was not downgraded because all estimates favoured yoga, heterogeneity was low to moderate (τ_study = 0.21), and the 95% prediction interval remained below the null (−1.17 to −0.09). Indirectness was not downgraded. Although 20 of 28 trials were conducted in India, the population, intervention, comparator, and objective outcome matched the review question directly, the mechanisms by which yoga affects glycaemia are not population-specific, and the India versus non-India estimates were concordant with no evidence of effect modification. Geographic concentration was therefore treated as a limitation of applicability, addressed in Section 4.4, rather than as indirectness. Imprecision was not downgraded because the pooled credible interval excluded the null with the point estimate exceeding the 0.5% threshold. Publication bias was downgraded one level because the PET-PEESE conditional estimate was attenuated and crossed the null (−0.32%, 95% CI −0.73 to 0.10), so small-study effects could not be excluded, even though Egger’s and Begg’s tests were non-significant and trim-and-fill estimates remained below zero. Final certainty was low (⊕⊕⚪⚪).

For FBG, certainty was downgraded one level for risk of bias and one level for suspected publication bias, giving low certainty. The risk-of-bias rationale mirrored that for HbA1c, with 23 of 24 studies carrying some concerns and one at high risk, and with the estimate essentially unchanged after excluding the high-risk study (−1.34 mmol/L, 95% CrI −1.73 to −0.97). Publication bias was downgraded because Egger’s test indicated asymmetry (p < 0.001) and trim-and-fill and PET-PEESE both yielded estimates smaller than the observed effect (−1.24 to −1.27 mmol/L), consistent with small-study effects. Inconsistency and imprecision were considered together, because the 95% prediction interval was wide and its upper bound (−0.03 mmol/L) lay close to the null. Imprecision was not downgraded because the pooled credible interval excluded the null and its least-benefit bound coincided with the 1.0 mmol/L threshold, so the pooled estimate was precise and clinically important. Inconsistency was not downgraded because the direction of effect was uniform (pd = 100%), the prediction interval remained below the null, and part of the between-study heterogeneity was explained by baseline FBG as an identified moderator. The wide prediction interval is reported as a limitation on generalisability in Section 4.4. Final certainty was low (⊕⊕⚪⚪).

## 4 Discussion

### 4.1 Principal findings

Pooling 28 RCTs and 2, 241 adults with T2DM, yoga reduced HbA1c by an average of 0.64% (95% CrI −0.87 to −0.41) and fasting plasma glucose by an average of 1.36 mmol/L (95% CrI −1.75 to −1.00) relative to usual care or waiting-list control. Direction probabilities reached 100% for both outcomes, and the model-level Bayes factors exceeded 1.2 × 10^4^ for HbA1c and 4.2 × 10^5^ for FBG, providing extreme evidence against the null hypothesis. Baseline glycemic level emerged as a credible moderator for both outcomes, with larger benefits at higher baseline values. An exploratory nonlinear association was observed between session duration and FBG. Studies with shorter sessions tended to report greater reductions. However, this finding was based on between-study comparisons and only seven distinct duration values. It may also reflect differences in study size, methodological quality, adherence, and related intervention characteristics. Therefore, it does not establish that shorter yoga sessions are more effective or define an optimal session duration. Under the GRADE framework, the certainty of evidence was low for both HbA1c and FBG, with downgrading driven by risk of bias and by suspected publication bias for each outcome. Secondary publication bias analyses supported the direction of both findings but showed different degrees of attenuation. For HbA1c, Egger’s and Begg’s tests were non-significant and trim-and-fill estimates remained below zero, but the PET-PEESE conditional estimate was attenuated and crossed the null. For FBG, all adjusted estimates remained below zero yet smaller than the observed effect. Both pooled effects may therefore be modestly overestimated, with the clearer small-study signal for FBG. For HbA1c, the bias-adjusted estimate could not be distinguished from the null under PET-PEESE, so small-study effects could not be excluded, and this discordance was the basis for rating HbA1c as low rather than moderate certainty.

### 4.2 Comparison with previous evidence

Our pooled estimates align in direction with earlier yoga meta-analyses but differ in magnitude. Earlier reviews reported HbA1c reductions of approximately 0.4%–0.5% (9, 12) smaller than our pooled estimate of 0.64%, a difference that probably reflects the growing number of eligible trials (22 vs. 7–14) together with a gradual increase in intervention dose across the literature. More recent yoga meta-analyses (10, 11, 42) have produced pooled estimates close to ours. A recently published Asia-focused meta-analysis (14) is worth contrasting. By dichotomizing total intervention duration into a contrast of fewer than 12 weeks versus 12 weeks or more, it concluded that HbA1c was reduced only when interventions lasted at least 12 weeks. In our Bayesian three-level framework, by contrast, continuous meta-regression on intervention duration did not identify weeks of practice as a credible moderator. This pattern suggests that dichotomized cut-off analyses may obscure the underlying continuous dose relationship.

When set against meta-analyses of general exercise modalities, the 0.64% HbA1c reduction observed here falls within the 0.4%–0.7% range reported for the main exercise modes (aerobic, resistance, and combined training) (43–45), indicating that the glycemic effect of yoga, as a mind-body practice, is comparable to that of conventional exercise. What distinguishes yoga from purely physical activity is its multi-pathway nature. Posture practice involves active muscular contraction and may promote translocation and expression of skeletal muscle glucose transporter type 4 (GLUT4) (46). Yoga RCTs have additionally documented improvements in the homeostatic model assessment for insulin resistance (HOMA-IR) (47), together with possible modulation of inflammatory markers (48). Pranayama and meditation can modulate vagal tone and improve heart rate variability (49), and may attenuate stress-induced insulin resistance through reductions in chronic stress and cortisol (50, 51). Given the substantial burden of depression and anxiety in T2DM populations (52, 53), yoga as a mind-body intervention may additionally improve glycemic control indirectly by relieving psychological comorbidity (54, 55). This indirect pathway may be especially relevant for specific anatomical phenotypes. Mitral valve prolapse and a narrow anteroposterior thoracic conformation are frequently accompanied by autonomic dysregulation, and have been associated with anxiety, although the strength of this link remains debated (56, 57). Patients with these characteristics may be more susceptible to stress-related hyperglycemia and could plausibly derive additional benefit from the anxiolytic and autonomic effects of yoga, a hypothesis that could not be tested in the present synthesis and warrants future prospective study. The convergence of physical, respiratory, and psychological mechanisms may explain why yoga produces an effect comparable to that of single-modality exercise. It also supports yoga as a feasible adjunctive option for patients with exercise-related fear, psychological comorbidity, or a preference for yoga.

Methodologically, the present analysis used a Bayesian three-level model to accommodate dependency arising from multiple arms or repeated time points within the same study (13), and followed the recommendation of IntHout et al. (15) to report 95% prediction intervals alongside credible intervals. The HbA1c prediction interval lay entirely below zero, indicating that the direction of effect is likely to be preserved across future settings. The FBG prediction interval ranged from −2.72 to −0.03 mmol/L, with the upper bound close to the null. Future studies may therefore find only small reductions, and the clinical benefit may be minimal in some settings despite a favorable direction of effect. Fitting both linear and spline forms to seven continuous moderators and selecting between them by LOO-IC allowed the analysis to identify two nonlinear moderators, baseline glycemic level and session duration, that might have been missed under a linear-only framework.

### 4.3 Clinical implications and exercise prescription

The reductions in HbA1c and FBG observed in this analysis exceed the thresholds commonly regarded as clinically meaningful (0.5% for HbA1c and 1.0 mmol/L for FBG) (2, 58). Long-term follow-up data from the UK Prospective Diabetes Study (UKPDS) (2) and the Atherosclerosis Risk in Communities (ARIC) cohort (59) further indicate that reductions of this size can translate into substantial microvascular and cardiovascular benefit over time. These findings may inform individualized practice, but they do not establish an optimal yoga dose. **Table 2** summarizes cautious practice considerations based on the available evidence.

**Table 2:** Yoga practice considerations for adults with type 2 diabetes.

Prescription parameter	Suggested approach	Supporting evidence from this meta-analysis	Evidence within this review
Yoga style	Hatha or composite yoga programmes (asana + pranayama ± meditation)	Yoga style was not formally tested as a moderator because the available studies were heavily unbalanced across styles; Hatha and composite programmes were the most frequently studied formats. Style-specific superiority cannot be established from current evidence.	Limited
Intensity	Light-to-moderate (≈ 2.5 METs)	75% of included studies used yoga practices in the 2.0–3.3 MET range (2024 Adult Compendium of Physical Activities). Insufficient between-study variation to test alternative intensities directly.	Limited
Frequency	3–5 sessions per week	Median across studies: 3 sessions/week (range 0.5–7). Meta-regression showed no significant linear effect of frequency on either outcome (HbA1c β = −0.12, 95% CrI −0.31 to +0.08; FBG β = −0.19, 95% CrI −0.60 to +0.20).	Limited
Session duration	No optimal session duration can currently be recommended. Duration should be individualized based on physical capacity, safety, feasibility, preference, and expected adherence	Median 60 min (range 10–90). For HbA1c, session duration showed no credible effect. For FBG, this exploratory finding was non-randomized, was based on only seven distinct duration values, and was partly driven by a single high-risk trial and by confounding with weekly frequency and dose. Within these limits, meta-regression suggested larger reductions with shorter sessions (20–30 min) and attenuated effects with longer sessions (≥60 min). This direction is opposite to usual exercise dose–response expectations and should not be read as “shorter is better”.	Limited
Programme duration	≥ 12 weeks (minimum 8 weeks)	Median 12 weeks (range 4–24). Most evidence clustered around 8–12 week programmes. Meta-regression showed no significant effect of programme weeks on either outcome, and the minimum effective duration remains uncertain.	Limited
Weekly dose	Individualize the weekly yoga dose based on feasibility, safety, and adherence. Current evidence does not support a fixed target of at least 600 MET-minutes per week.	HbA1c effects were identical above and below 600 MET minutes per week. FBG reductions were greater above this threshold, but the credible intervals overlapped. The Bayes factor showed no evidence of effect modification, although this comparison was underpowered. This threshold was derived from general physical activity guidelines and has not been validated as an optimal yoga dose.	Limited
Supervision	Supervised group sessions, with or without home practice	Subgroup estimates were identical for supervised-only and supervised-plus-home programmes in the FBG subset (−1.36 vs −1.36 mmol/L, interaction BF_10_ = 0.19). This comparison was underpowered, so it indicates an absence of evidence for effect modification rather than established equivalence. Both delivery modes appear usable.	Limited
Suitable population	Adults with established type 2 diabetes; greater benefit in those with higher baseline values	Baseline HbA1c moderated treatment effect (β = −0.20, 95% CrI −0.39 to −0.02), as did baseline FBG (β = −0.49, 95% CrI −0.85 to −0.15): patients with poorer baseline glycaemic control derived larger absolute reductions.	Well-supported
Expected effect size	HbA1c: ≈ 0.5–0.7% reduction FBG: ≈ 1.0–1.5 mmol/L reduction	Pooled estimates: HbA1c −0.64% (95% CrI −0.87 to −0.41); FBG −1.36 mmol/L (95% CrI −1.75 to −1.00). Both exceed commonly cited clinical thresholds (HbA1c ≥ 0.5%; FBG ≥ 1.0 mmol/L).	Well-supported
Adjunct status	Use as an adjunct to standard medical care, not a replacement	Most trials evaluated yoga as an adjunct to ongoing diabetes care. Medication classes and dose stability were often incompletely reported, and a few trials showed baseline imbalances or differential dose changes.	Limited

These practice considerations are derived directly from the present three-level Bayesian meta-analysis of 28 randomised controlled trials and are intended to support individualised clinical judgement rather than fixed prescriptions. The final column describes how well each consideration is supported by the evidence within this review. Well-supported denotes a consideration backed by a credible interval excluding zero, a concordant pattern across the included trials, or near-universal practice across trials. Limited denotes a consideration based on limited, indirect, unbalanced, or exploratory evidence. This column is distinct from the GRADE certainty of evidence, which was low for both HbA1c and FBG (**Table 1**), and it does not represent a formal GRADE strength of recommendation. METs refer to metabolic equivalents from the 2024 Adult Compendium of Physical Activities (Herrmann et al., 2024). WHO threshold refers to the World Health Organization 2020 physical activity guidelines for adults (at least 600 MET-minutes per week of moderate-intensity activity). Clinical thresholds for meaningful change are 0.5% for HbA1c (per ADA Standards of Care) and 1.0 mmol/L for fasting glucose. CrI, credible interval. BF_10_, Bayes factor for the interaction model over the additive model.

With respect to patient selection, the magnitude of benefit increased monotonically with baseline glycemic level, indicating that yoga should be considered preferentially for patients whose glucose control is suboptimal or who remain above target at initial diagnosis. This baseline-dependent efficacy profile is among the most clinically valuable attributes of yoga as an adjunctive therapy. The meta-regression did not provide a reliable basis for identifying an optimal session duration. The association between shorter sessions and greater FBG reductions was based on between-study comparisons rather than trials that directly compared different durations. It included only seven distinct duration values and may have been confounded by study size, risk of bias, adherence, baseline glycemia, weekly frequency, yoga intensity, program duration, and other study characteristics. The trial with the shortest sessions included only 10 participants and was the only FBG study rated at high overall risk of bias. Adherence was also reported inconsistently, making it unclear whether the prescribed duration reflected the actual dose completed. This finding should therefore be considered hypothesis-generating and does not establish that shorter sessions are more effective. Until direct dose-comparison trials are available, session duration should be individualized based on physical capacity, safety, treatment goals, preferences, feasibility, and expected adherence.

Once a dose has been selected, the mode of delivery can remain flexible. Supervision was not a credible moderator in our analysis, and the fully supervised and supervised-plus-home strata produced almost identical effects in the FBG subset. This comparison was underpowered and does not prove equivalence, but it leaves room for adaptation when outpatient capacity is limited or when patients cannot attend on-site sessions frequently. A practical pathway is to begin with supervised practice to ensure correct technique, then transition gradually to home-based self-management. For the choice of yoga style, no single style proved superior in our analysis, and clinicians and exercise specialists can integrate cultural acceptability, physical condition, and individual preference into their recommendations. Hatha yoga and composite yoga programs carry the most extensive evidence base. Protocols emphasizing pranayama may yield greater circulatory and stress-related benefits, whereas more vigorous styles approach the metabolic profile of conventional exercise more closely.

### 4.4 Strengths and limitations

The principal strengths of this review lie in the use of a Bayesian three-level model combined with spline meta-regression, in the separate appraisal of risk of bias for each outcome, and in the inclusion of comprehensive leave-one-out and pre–post correlation sensitivity analyses. Together, these features increase methodological rigor relative to earlier yoga meta-analyses.

Several limitations warrant careful consideration. No study was at overall low risk for either outcome. Most had some concerns, while two HbA1c studies and one FBG study were at high risk. Concerns involved randomization, deviations from intended interventions, missing data, and selective reporting. Excluding high risk studies did not materially change the pooled estimates, supporting robustness but not eliminating the remaining limitations. GRADE certainty was therefore downgraded one level for risk of bias for both outcomes. For HbA1c, Egger’s and Begg’s tests did not indicate funnel plot asymmetry, and trim-and-fill estimates remained below zero. The PET-PEESE conditional estimate, however, was attenuated and crossed the null, so small-study effects could not be excluded. HbA1c was therefore downgraded one level for suspected publication bias. This was a conservative decision that reflects the discordance among methods rather than confirmed bias. Egger’s test indicated funnel plot asymmetry for FBG. Trim and fill and PET PEESE analyses yielded smaller estimates, but all remained below zero. This suggests that small study effects may have inflated the pooled magnitude without changing its direction. However, these methods rely on assumptions linking study precision to effect size and cannot confirm the presence or extent of publication bias. FBG was downgraded one additional level for suspected publication bias. Background pharmacotherapy was poorly reported. Only six trials clearly documented no medication changes in either group during follow up. Several reported only broad drug classes or baseline treatment stability, while others omitted medication details. One trial had a marked baseline imbalance in metformin use, and another reported more dose reductions in the yoga group. These changes may have reduced the between group contrast or reflected improved glycemic control. Residual confounding from concomitant therapy cannot be excluded. The data were too sparse and heterogeneous for medication based subgroup analysis or meta regression. External validity is limited because 20 of the 28 trials were conducted in India. Although estimates were similar across regions, only six non-Indian studies contributed to the HbA1c analysis and five to the FBG analysis. This limited power to detect geographic differences, and the absence of interaction does not establish cross-cultural equivalence. The findings are therefore most applicable to Indian settings. More trials from other regions are needed to support broader generalization. The median intervention duration was only 12 weeks (maximum 24 weeks), leaving direct evidence largely unavailable for both long-term maintenance and hard endpoints such as cardiovascular events and microvascular complications. In addition, incomplete reporting of yoga intervention content limited inferences about specific styles and component combinations. MET values were assigned from the Compendium based on reported intervention content. Incomplete reporting may have caused exposure misclassification and reduced the accuracy of the dose response analyses. The session duration meta-regression was also based on aggregate data and only seven distinct values, making it vulnerable to ecological bias and residual confounding. Inconsistent adherence reporting further limited assessment of the actual dose completed. The apparent benefit of shorter sessions should therefore be considered exploratory and should not be used to establish causality or define an optimal duration.

## 5 Conclusion

Yoga is a feasible adjunctive option for glycemic management in adults with T2DM, with pooled effect sizes comparable in magnitude to those of mainstream exercise modalities and the added value of physical, respiratory, and psychological pathways of benefit, although the certainty of this evidence is low. Of clinical relevance, trials enrolling participants with poorer baseline glycemic control showed larger effects, indicating particular value for those whose glucose remains suboptimally controlled. Current evidence neither identifies an optimal yoga session duration nor shows that shorter sessions are superior. Session duration should therefore be individualized until adequately powered trials directly compare different prescriptions. With respect to the evidence base, certainty was low for both HbA1c and FBG, owing to risk of bias and suspected publication bias. The pooled effects remained favourable and directionally consistent across all bias-adjusted and sensitivity analyses, although one bias-adjusted analysis left the HbA1c benefit indistinguishable from the null. Yoga can therefore be considered as an adjunctive option within comprehensive T2DM management, with the low certainty of evidence made explicit. Because most trials were conducted in India, the applicability of these findings to other cultural and health care settings remains uncertain. Future trials should improve intervention reporting, recruit more diverse populations, and extend follow-up to clarify the role of yoga across clinical and cultural settings.

## Data Availability

The original contributions presented in the study are included in the article/**Supplementary Material**. Further inquiries can be directed to the corresponding author.

## References

[1] International Diabetes Federation . Idf Diabetes Atlas. Brussels, Belgium: International Diabetes Federation (2025).

[2] Stratton IM Adler AI Neil HAW Matthews DR Manley SE Cull CA . Association of glycaemia with macrovascular and microvascular complications of type 2 diabetes (UKPDS 35): prospective observational study. Bmj. (2000) 321:405–12. doi: 10.1136/bmj.321.7258.405 10938048 PMC27454

[3] Piragine E Petri D Martelli A Calderone V Lucenteforte E . Adherence to oral antidiabetic drugs in patients with type 2 diabetes: systematic review and meta-analysis. J Clin Med. (2023) 12:1981. doi: 10.3390/jcm12051981 36902770 PMC10004070

[4] Nong K Jeppesen BT Shi Q Agoritsas T Guyatt GH White H . Medications for adults with type 2 diabetes: a living systematic review and network meta-analysis. Bmj. (2025) 390. doi: 10.1136/bmj-2024-083039 40813122

[5] Bull FC Al-Ansari SS Biddle S Borodulin K Buman MP Cardon G . World Health Organization 2020 guidelines on physical activity and sedentary behaviour. Br J Sports Med. (2020) 54:1451–62. doi: 10.1136/bjsports-2020-102955 33239350 PMC7719906

[6] Kanaley JA Colberg SR Corcoran MH Malin SK Rodriguez NR Crespo CJ . Exercise/physical activity in individuals with type 2 diabetes: a consensus statement from the American College of Sports Medicine. Med Sci Sports Exercise. (2022) 54:353. doi: 10.1249/mss.0000000000002800 35029593 PMC8802999

[7] American Diabetes Association Professional PracticeCommittee for Diabetes . Facilitating positive health behaviors and well-being to improve health outcomes: Standards of care in diabetes-2026. Diabetes Care. (2026) 49:S89–S131. doi: 10.2337/dc26-s005 41358898 PMC12690188

[8] Raveendran AV Deshpandae A Joshi SR . Therapeutic role of yoga in type 2 diabetes. Endocrinol Metab. (2018) 33:307. doi: 10.3803/enm.2018.33.3.307 30112866 PMC6145966

[9] Innes KE Selfe TK . Yoga for adults with type 2 diabetes: a systematic review of controlled trials. J Diabetes Res. (2016) 2016:6979370. doi: 10.1155/2016/6979370 26788520 PMC4691612

[10] Thind H Lantini R Balletto BL Donahue ML Salmoirago-Blotcher E Bock BC . The effects of yoga among adults with type 2 diabetes: a systematic review and meta-analysis. Prev Med. (2017) 105:116–26. doi: 10.1016/j.ypmed.2017.08.017 28882745 PMC5653446

[11] Chen S Deng S Liu Y Yin T . Effects of yoga on blood glucose and lipid profile of type 2 diabetes patients without complications: a systematic review and meta-analysis. Front Sports Active Living. (2022) 4:900815. doi: 10.3389/fspor.2022.900815 35813055 PMC9259958

[12] Cui J Yan JH Yan LM Pan L Le JJ Guo YZ . Effects of yoga in adults with type 2 diabetes mellitus: a meta-analysis. J Diabetes Invest. (2017) 8:201–9. doi: 10.1111/jdi.12548 27370357 PMC5334310

[13] Van den Noortgate W López-López JA Marín-Martínez F Sánchez-Meca J . Three-level meta-analysis of dependent effect sizes. Behav Res Methods. (2013) 45:576–94. doi: 10.3758/s13428-012-0261-6 23055166

[14] Mahajan A Shah J Muley A . Yoga practice duration and its influence on type 2 diabetes management: a meta-analysis of Asian population. iScience. (2026) 29:115288. doi: 10.1016/j.isci.2026.115288 42006380 PMC13090653

[15] IntHout J Ioannidis JP Rovers MM Goeman JJ . Plea for routinely presenting prediction intervals in meta-analysis. BMJ Open. (2016) 6:e010247. doi: 10.1136/bmjopen-2015-010247 27406637 PMC4947751

[16] Page MJ McKenzie JE Bossuyt PM Boutron I Hoffmann TC Mulrow CD . The PRISMA 2020 statement: an updated guideline for reporting systematic reviews. Bmj. (2021) 372:n71. doi: 10.1136/bmj.n71 33782057 PMC8005924

[17] Chandler J Cumpston M Li T Page MJ Welch V . Cochrane Handbook for Systematic Reviews of Interventions Vol. 4. . Hoboken: Wiley (2019). p. 14651858.

[18] Hoelzel W Weykamp C Jeppsson J-O Miedema K Barr JR Goodall I . IFCC reference system for measurement of hemoglobin A1c in human blood and the national standardization schemes in the United States, Japan, and Sweden: a method-comparison study. Clin Chem. (2004) 50:166–74. doi: 10.1373/clinchem.2003.024802 14709644

[19] Follmann D Elliott P Suh I Cutler J . Variance imputation for overviews of clinical trials with continuous response. J Clin Epidemiol. (1992) 45:769–73. doi: 10.1016/0895-4356(92)90054-q 1619456

[20] Lenters-Westra E Schindhelm RK Bilo HJG Groenier KH Slingerland RJ . Differences in interpretation of haemoglobin A1c values among diabetes care professionals. Neth J Med. (2014) 72(9):462–6. 25431391

[21] Dong C Liu R Huang Z Yang Y Sun S Li R . Effect of exercise interventions based on family management or self-management on glycaemic control in patients with type 2 diabetes mellitus: a systematic review and meta-analysis. Diabetol Metab Syndrome. (2023) 15:232. doi: 10.1186/s13098-023-01209-4 37957775 PMC10644553

[22] Herrmann SD Willis EA Ainsworth BE Barreira TV Hastert M Kracht CL . 2024 Adult Compendium of Physical Activities: a third update of the energy costs of human activities. J Sport Health Sci. (2024) 13:6–12. doi: 10.1016/j.jshs.2023.10.010 38242596 PMC10818145

[23] Sterne JA Savović J Page MJ Elbers RG Blencowe NS Boutron I . RoB 2: a revised tool for assessing risk of bias in randomised trials. Bmj. (2019) 366:l4898. doi: 10.1136/bmj.l4898 31462531

[24] McGuinness LA Higgins JP . Risk‐of‐bias VISualization (robvis): an R package and Shiny web app for visualizing risk‐of‐bias assessments. Res Synth Methods. (2021) 12:55–61. doi: 10.1002/jrsm.1411 32336025

[25] Cheung M-L . Modeling dependent effect sizes with three-level meta-analyses: a structural equation modeling approach. psychol Methods. (2014) 19:211. doi: 10.1037/a0032968 23834422

[26] Assink M Wibbelink CJ . Fitting three-level meta-analytic models in R: a step-by-step tutorial. Quantitative Methods For Psychol. (2016) 12:154–74. doi: 10.20982/tqmp.12.3.p154

[27] Röver C Bender R Dias S Schmid CH Schmidli H Sturtz S . On weakly informative prior distributions for the heterogeneity parameter in Bayesian random‐effects meta‐analysis. Res Synth Methods. (2021) 12:448–74. doi: 10.1002/jrsm.1475 33486828

[28] Bürkner P-C . brms: an R package for Bayesian multilevel models using Stan. J Stat Software. (2017) 80:1–28. doi: 10.18637/jss.v080.i01

[29] Carpenter B Gelman A Hoffman MD Lee D Goodrich B Betancourt M . Stan: a probabilistic programming language. J Stat Software. (2017) 76:1–32. doi: 10.18637/jss.v076.i01 36568334 PMC9788645

[30] Hoffman MD Gelman A . The No-U-Turn sampler: adaptively setting path lengths in Hamiltonian Monte Carlo. J Mach Learn Res. (2014) 15:1593–623. doi: 10.5555/2627435.2638586

[31] Vehtari A Gelman A Simpson D Carpenter B Bürkner P-C . Rank-normalization, folding, and localization: an improved $\hat{R}$ for assessing convergence of MCMC (with discussion). Bayesian Anal. (2021) 16:667–718. doi: 10.1214/20-ba1221 42159763

[32] Makowski D Ben-Shachar MS Lüdecke D . bayestestR: describing effects and their uncertainty, existence and significance within the Bayesian framework. J Open Source Software. (2019) 4:1541. doi: 10.21105/joss.01541

[33] Gronau QF Singmann H Wagenmakers E-J . bridgesampling: an R package for estimating normalizing constants. J Stat Software. (2020) 92:1–29. doi: 10.31222/osf.io/v94h6 42226319

[34] Kass RE Raftery AE . Bayes factors. J Am Stat Assoc. (1995) 90:773–95. doi: 10.2307/2348679

[35] Schielzeth H . Simple means to improve the interpretability of regression coefficients. Methods Ecol Evol. (2010) 1:103–13. doi: 10.1111/j.2041-210x.2010.00012.x 40046247

[36] Vehtari A Gelman A Gabry J . Practical Bayesian model evaluation using leave-one-out cross-validation and WAIC. Stat Computing. (2017) 27:1413–32. doi: 10.1007/s11222-016-9696-4 30311153

[37] Viechtbauer W . Conducting meta-analyses in R with the metafor package. J Stat Software. (2010) 36:1–48. doi: 10.18637/jss.v036.i03

[38] Peters JL Sutton AJ Jones DR Abrams KR Rushton L . Contour-enhanced meta-analysis funnel plots help distinguish publication bias from other causes of asymmetry. J Clin Epidemiol. (2008) 61:991–6. doi: 10.1016/j.jclinepi.2007.11.010 18538991

[39] Egger M Smith GD Schneider M Minder C . Bias in meta-analysis detected by a simple, graphical test. Bmj. (1997) 315:629–34. doi: 10.1136/bmj.315.7109.629 9310563 PMC2127453

[40] Begg CB Mazumdar M . Operating characteristics of a rank correlation test for publication bias. Biometrics. (1994), 1088–101. doi: 10.2307/2533446 7786990

[41] Guyatt GH Oxman AD Vist GE Kunz R Falck-Ytter Y Alonso-Coello P . GRADE: an emerging consensus on rating quality of evidence and strength of recommendations. Bmj. (2008) 336:924–31. doi: 10.1136/bmj.39489.470347.ad 18436948 PMC2335261

[42] JavedD ChandraMB MalhotraV MuddaS RP KarrothuVV . The impact of yoga as an adjunct to standard care on glycemic control, insulin resistance, oxidative stress, and quality of life in individuals with or at risk of type 2 diabetes mellitus: a systematic review and meta-analysis. Curr Diabetes Rev. (2026) 22:e15733998383494. doi: 10.2174/0115733998383494250827215758 40993952

[43] Hou L Wang Q Pan B Li R Li Y He J . Exercise modalities for type 2 diabetes: a systematic review and network meta-analysis of randomized trials. Diabetes/Metabolism Res Rev. (2023) 39:e3591. doi: 10.1002/dmrr.3591 36423199

[44] Garcia SP Cureau FV Iorra FQ Bottino LG Monteiro LERC Leivas G . Effects of exercise training and physical activity advice on HbA1c in people with type 2 diabetes: a network meta-analysis of randomized controlled trials. Diabetes Res Clin Pract. (2025) 221:112027. doi: 10.1016/j.diabres.2025.112027 39904457

[45] Michielsen M Yagiz J Hanssens M Claes J Geuns L Gojevic T . The effect of exercise characteristics on HbA1c and other cardiovascular risk factors in adults with type 2 diabetes: a systematic review and meta-analysis of randomised controlled trials. Cardiovasc Diabetol. (2025) 25:30. doi: 10.1186/s12933-025-03048-1 41457265 PMC12859889

[46] Richter EA Hargreaves M . Exercise, GLUT4, and skeletal muscle glucose uptake. Physiol Rev. (2013) 93:993–1017. doi: 10.1152/physrev.00038.2012 23899560

[47] Dhali B Chatterjee S Das S Cruz M . Effect of yoga on insulin resistance in type 2 diabetes: a systematic review and meta-analysis. Clin Diabetol. (2023) 12:201–8. doi: 10.5603/dk.a2023.0022

[48] Mishra B Agarwal A George JA Upadhyay AD Nilima N Mishra R . Effectiveness of yoga in modulating markers of immunity and inflammation: a systematic review and meta-analysis. Cureus. (2024) 16:e57541. doi: 10.7759/cureus.57541 38707001 PMC11068076

[49] Danasegaran M Pal GK Sahoo J Pal P Nanda N Renugasundari M . Effects of 12 weeks practice of yoga on heart rate variability in males with type 2 diabetes receiving oral antidiabetic drugs: a randomized control trial. J Altern Complementary Med (New York NY). (2021) 27:1105–15. doi: 10.1089/acm.2020.0489 34582701

[50] Begg CB Mazumdar M . Operating characteristics of a rank correlation test for publication bias. Biometrics. (1994) 50(4):1088–101. doi: 10.2307/2533446 7786990

[51] Schleinzer A Moosburner A Anheyer D Burgahn L Cramer H . Effects of yoga on stress in stressed adults: a systematic review and meta-analysis. Front Psychiatry. (2024) 15:1437902. doi: 10.3389/fpsyt.2024.1437902 39553891 PMC11563964

[52] Farooqi A Gillies C Sathanapally H Abner S Seidu S Davies MJ . A systematic review and meta-analysis to compare the prevalence of depression between people with and without Type 1 and Type 2 diabetes. Primary Care Diabetes. (2022) 16:1–10. doi: 10.1016/j.pcd.2021.11.001 34810141

[53] Yang K Fang Y He J Li J . Prevalence and risk factors of depression in patients with diabetes mellitus: a systematic review and meta-analysis. Front Endocrinol. (2025) 16:1660478. doi: 10.3389/fendo.2025.1660478 41189617 PMC12580098

[54] Ee CC Al-Kanini I Armour M Piya MK McMorrow R Rao VS . Mindfulness-based interventions for adults with type 2 diabetes mellitus: a systematic review and meta-analysis. Integr Med Res. (2025) 14:101138. doi: 10.1016/j.imr.2025.101138 40386581 PMC12084514

[55] Tang Y Zhang Y Su H Lv Y Sun M Yu L . High-frequency, short-session exercise decreases anxiety and depression in individuals with type 2 diabetes mellitus: a systematic review and meta-analysis of randomized controlled trials. Behav Sci (Basel Switzerland). (2026) 16:15. doi: 10.3390/bs16010015 41594958 PMC12837154

[56] Sonaglioni A Nicolosi GL Lombardo M . The relationship between mitral valve prolapse and thoracic skeletal abnormalities in clinical practice: a systematic review. J Cardiovasc Med (Hagerstown Md). (2024) 25:353–63. doi: 10.2459/jcm.0000000000001614 38526955

[57] Tural U Iosifescu DV . The prevalence of mitral valve prolapse in panic disorder: a meta-analysis. Psychosomatics. (2019) 60:393–401. doi: 10.1016/j.psym.2018.10.002 30448200

[58] American Diabetes Association Professional PracticeCommittee for Diabetes . Diagnosis and classification of diabetes: standards of care in diabetes-2026. Diabetes Care. (2026) 49:S27–49. doi: 10.2337/dc26-s002 41358893 PMC12690183

[59] Selvin E Steffes MW Zhu H Matsushita K Wagenknecht L Pankow J . Glycated hemoglobin, diabetes, and cardiovascular risk in nondiabetic adults. N Engl J Med. (2010) 362:800–11. doi: 10.1056/nejmoa0908359 20200384 PMC2872990

