# Supplementary material for: Effect of yoga on glycemic control in adults with type 2 diabetes mellitus: a Bayesian three-level meta-analysis of randomized controlled trials
Source: Front Endocrinol (Lausanne). 2026 Aug 11;17:1889124. doi: 10.3389/fendo.2026.1889124 (PMC13503593; doi:10.3389/fendo.2026.1889124)
Supplement: Supplementary file 1 [file Supplementaryfile1.docx]

| **Section and Topic** | **Item #** | **Checklist item** | **Location where item is reported** |
| --- | --- | --- | --- |
| **TITLE** | | |  |
| Title | 1 | Identify the report as a systematic review. | Title page (manuscript title) |
| **ABSTRACT** | | |  |
| Abstract | 2 | See the PRISMA 2020 for Abstracts checklist. | Abstract (Background / Methods / Results / Conclusions structured abstract) |
| **INTRODUCTION** | | |  |
| Rationale | 3 | Describe the rationale for the review in the context of existing knowledge. | Section 1 Introduction (paragraphs 1–3) |
| Objectives | 4 | Provide an explicit statement of the objective(s) or question(s) the review addresses. | Section 1 Introduction (final paragraph, study aims) |
| **METHODS** | | |  |
| Eligibility criteria | 5 | Specify the inclusion and exclusion criteria for the review and how studies were grouped for the syntheses. | Section 2.3 Eligibility criteria (PICOS sub-paragraphs) |
| Information sources | 6 | Specify all databases, registers, websites, organisations, reference lists and other sources searched or consulted to identify studies. Specify the date when each source was last searched or consulted. | Section 2.2 Search strategy |
| Search strategy | 7 | Present the full search strategies for all databases, registers and websites, including any filters and limits used. | Section 2.2 Search strategy; Supplementary File 1 (full search strategies) |
| Selection process | 8 | Specify the methods used to decide whether a study met the inclusion criteria of the review, including how many reviewers screened each record and each report retrieved, whether they worked independently, and if applicable, details of automation tools used in the process. | Section 2.4 Study selection and data extraction |
| Data collection process | 9 | Specify the methods used to collect data from reports, including how many reviewers collected data from each report, whether they worked independently, any processes for obtaining or confirming data from study investigators, and if applicable, details of automation tools used in the process. | Section 2.4 Study selection and data extraction |
| Data items | 10a | List and define all outcomes for which data were sought. Specify whether all results that were compatible with each outcome domain in each study were sought (e.g. for all measures, time points, analyses), and if not, the methods used to decide which results to collect. | Section 2.3 Eligibility criteria (Outcomes O); Section 2.5 Effect size measurement and dose coding |
|  | 10b | List and define all other variables for which data were sought (e.g. participant and intervention characteristics, funding sources). Describe any assumptions made about any missing or unclear information. | Section 2.4 Study selection and data extraction; Section 2.5 Effect size measurement and dose coding |
| Study risk of bias assessment | 11 | Specify the methods used to assess risk of bias in the included studies, including details of the tool(s) used, how many reviewers assessed each study and whether they worked independently, and if applicable, details of automation tools used in the process. | Section 2.6 Risk of bias assessment; Supplementary File 3 (outcome-specific RoB tables); Supplementary File 4 (RoB summary barplots) |
| Effect measures | 12 | Specify for each outcome the effect measure(s) (e.g. risk ratio, mean difference) used in the synthesis or presentation of results. | Section 2.5 Effect size measurement and dose coding; Section 2.7 Statistical analysis |
| Synthesis methods | 13a | Describe the processes used to decide which studies were eligible for each synthesis (e.g. tabulating the study intervention characteristics and comparing against the planned groups for each synthesis (item #5)). | Section 2.3 Eligibility criteria |
|  | 13b | Describe any methods required to prepare the data for presentation or synthesis, such as handling of missing summary statistics, or data conversions. | Section 2.5 Effect size measurement and dose coding; Section 2.7 Statistical analysis |
|  | 13c | Describe any methods used to tabulate or visually display results of individual studies and syntheses. | Section 2.7 Statistical analysis; Figures 2–5; Table 1; Supplementary Files 5–8 |
|  | 13d | Describe any methods used to synthesize results and provide a rationale for the choice(s). If meta-analysis was performed, describe the model(s), method(s) to identify the presence and extent of statistical heterogeneity, and software package(s) used. | Section 2.7 Statistical analysis (Bayesian three-level random-effects model) |
|  | 13e | Describe any methods used to explore possible causes of heterogeneity among study results (e.g. subgroup analysis, meta-regression). | Section 2.7 Statistical analysis (Subgroup analyses and meta-regression) |
|  | 13f | Describe any sensitivity analyses conducted to assess robustness of the synthesized results. | Section 2.7 Statistical analysis (Sensitivity analyses) |
| Reporting bias assessment | 14 | Describe any methods used to assess risk of bias due to missing results in a synthesis (arising from reporting biases). | Section 2.7 Statistical analysis (Publication bias) |
| Certainty assessment | 15 | Describe any methods used to assess certainty (or confidence) in the body of evidence for an outcome. | Section 2.7 Statistical analysis (GRADE) |
| **RESULTS** | | |  |
| Study selection | 16a | Describe the results of the search and selection process, from the number of records identified in the search to the number of studies included in the review, ideally using a flow diagram. | Section 3.1 Search results; Figure 1 (PRISMA 2020 flow diagram) |
|  | 16b | Cite studies that might appear to meet the inclusion criteria, but which were excluded, and explain why they were excluded. | Section 3.1 Search results (numbers and reasons for full-text exclusions); Figure 1 |
| Study characteristics | 17 | Cite each included study and present its characteristics. | Section 3.2 Characteristics of the included studies; Supplementary File 2 |
| Risk of bias in studies | 18 | Present assessments of risk of bias for each included study. | Section 3.3 Risk of bias; Supplementary File 3 (per-study RoB tables, by outcome); Supplementary File 4 (RoB summary barplots) |
| Results of individual studies | 19 | For all outcomes, present, for each study: (a) summary statistics for each group (where appropriate) and (b) an effect estimate and its precision (e.g. confidence/credible interval), ideally using structured tables or plots. | Section 3.4 Main analyses; Figure 2 (forest plots for both outcomes) |
| Results of syntheses | 20a | For each synthesis, briefly summarise the characteristics and risk of bias among contributing studies. | Section 3.4 Main analyses (HbA1c and FBG paragraphs) |
|  | 20b | Present results of all statistical syntheses conducted. If meta-analysis was done, present for each the summary estimate and its precision (e.g. confidence/credible interval) and measures of statistical heterogeneity. If comparing groups, describe the direction of the effect. | Section 3.4 Main analyses; Figure 2; Table 1 |
|  | 20c | Present results of all investigations of possible causes of heterogeneity among study results. | Section 3.5 Subgroup analyses (Figure 3); Section 3.6 Meta-regression (Figures 4 and 5); Supplementary File 7 |
|  | 20d | Present results of all sensitivity analyses conducted to assess the robustness of the synthesized results. | Section 3.8 Sensitivity analyses; Supplementary Files 10–13 |
| Reporting biases | 21 | Present assessments of risk of bias due to missing results (arising from reporting biases) for each synthesis assessed. | Section 3.7 Publication bias; Supplementary File 8 (funnel plots); Supplementary File 9 (Egger and Begg test results) |
| Certainty of evidence | 22 | Present assessments of certainty (or confidence) in the body of evidence for each outcome assessed. | Section 3.9 Certainty of evidence; Table 1 (GRADE summary) |
| **DISCUSSION** | | |  |
| Discussion | 23a | Provide a general interpretation of the results in the context of other evidence. | Section 4.1 Principal findings; Section 4.2 Comparison with previous evidence |
|  | 23b | Discuss any limitations of the evidence included in the review. | Section 4.4 Strengths and limitations |
|  | 23c | Discuss any limitations of the review processes used. | Section 4.4 Strengths and limitations |
|  | 23d | Discuss implications of the results for practice, policy, and future research. | Section 4.3 Clinical implications and exercise prescription; Section 4.5 Conclusion; Table 2 |
| **OTHER INFORMATION** | | |  |
| Registration and protocol | 24a | Provide registration information for the review, including register name and registration number, or state that the review was not registered. | Section 2.1 Study protocol and reporting standards (PROSPERO CRD 420261391309) |
|  | 24b | Indicate where the review protocol can be accessed, or state that a protocol was not prepared. | Section 2.1 Study protocol and reporting standards (protocol available on the PROSPERO record) |
|  | 24c | Describe and explain any amendments to information provided at registration or in the protocol. | Not applicable; no amendments were made after registration |
| Support | 25 | Describe sources of financial or non-financial support for the review, and the role of the funders or sponsors in the review. | Title page (Funding statement) |
| Competing interests | 26 | Declare any competing interests of review authors. | Title page (Competing interests statement) |
| Availability of data, code and other materials | 27 | Report which of the following are publicly available and where they can be found: template data collection forms; data extracted from included studies; data used for all analyses; analytic code; any other materials used in the review. | Title page (Availability of data and materials statement); Supplementary Files 1–13 |

*From:*  Page MJ, McKenzie JE, Bossuyt PM, Boutron I, Hoffmann TC, Mulrow CD, et al. The PRISMA 2020 statement: an updated guideline for reporting systematic reviews. BMJ 2021;372:n71. doi: 10.1136/bmj.n71. This work is licensed under CC BY 4.0. To view a copy of this license, visit <https://creativecommons.org/licenses/by/4.0/>
