# Supplementary material for: Effect of yoga on glycemic control in adults with type 2 diabetes mellitus: a Bayesian three-level meta-analysis of randomized controlled trials
Source: Front Endocrinol (Lausanne). 2026 Aug 11;17:1889124. doi: 10.3389/fendo.2026.1889124 (PMC13503593; doi:10.3389/fendo.2026.1889124)
Supplement: Supplementary file 2 [file Supplementaryfile2.docx]

**Effect of yoga on glycemic control in adults with type 2 diabetes mellitus: a Bayesian three-level meta-analysis of randomized controlled trials**

**Supplementary Materials**

**Contents**

Supplementary File 1. Detailed search strategies for all four databases

Supplementary File 2. Characteristics of included studies and background pharmacotherapy

Supplementary File 3. Risk of Bias 2.0 assessment (by outcome)

Supplementary File 4. Risk of Bias 2.0 summary bar plots (by outcome)

Supplementary File 5. Posterior distributions of pooled effects and heterogeneity

Supplementary File 6. Posterior predictive checks

Supplementary File 7. Moderator analyses (subgroup + meta-regression)

Supplementary File 8. Funnel plots

Supplementary File 9. Egger and Begg publication-bias tests

Supplementary File 10. Leave-one-out sensitivity (numerical results)

Supplementary File 11. Leave-one-out sensitivity (visualisation)

Supplementary File 12. Pre-post correlation sensitivity (numerical)

Supplementary File 13. Pre-post correlation sensitivity (visualisation)

Supplementary File 14. Sensitivity analysis excluding studies at high overall risk of bias

Supplementary File 15. Publication bias adjustment for glycemic outcomes

Supplementary File 16. GRADE evidence profile.

**Supplementary File 1. Detailed search strategies**

Systematic searches were performed in four electronic databases (PubMed, Embase, Web of Science, and Cochrane Library) in May 2026. No date or language restrictions were applied at the search stage; language was assessed at the screening stage. Search terms combined controlled vocabulary (MeSH or Emtree) with free-text terms covering three conceptual blocks: (i) population (type 2 diabetes), (ii) intervention (yoga and related practices), and (iii) randomised study design.

**1.1 PubMed**

*Date run: May 2026. Total records retrieved: 157.*

| **Set** | **Search query** | **Records** |
| --- | --- | --- |
| **#1** | "Diabetes Mellitus, Type 2"[Mesh] OR "type 2 diabetes"[Title/Abstract] OR "type 2 diabetes mellitus"[Title/Abstract] OR T2DM[Title/Abstract] OR "non-insulin-dependent diabetes"[Title/Abstract] OR "non insulin dependent diabetes"[Title/Abstract] | Population |
| **#2** | "Yoga"[Mesh] OR yoga[Title/Abstract] OR yogic[Title/Abstract] OR asana[Title/Abstract] OR asanas[Title/Abstract] OR pranayama[Title/Abstract] OR "yoga therapy"[Title/Abstract] OR "therapeutic yoga"[Title/Abstract] | Intervention |
| **#3** | "Hemoglobin A, Glycosylated"[Mesh] OR HbA1c[Title/Abstract] OR A1c[Title/Abstract] OR "glycated hemoglobin"[Title/Abstract] OR "glycated haemoglobin"[Title/Abstract] OR "glycosylated hemoglobin"[Title/Abstract] OR "glycosylated haemoglobin"[Title/Abstract] OR "hemoglobin A1c"[Title/Abstract] OR "haemoglobin A1c"[Title/Abstract] OR "Blood Glucose"[Mesh] OR "fasting blood glucose"[Title/Abstract] OR "fasting plasma glucose"[Title/Abstract] OR FBG[Title/Abstract] OR FPG[Title/Abstract] | Outcome |
| **#4** | randomized controlled trial[Publication Type] OR controlled clinical trial[Publication Type] OR random*[Title/Abstract] OR randomly[Title/Abstract] OR trial[Title/Abstract] OR placebo[Title/Abstract] OR groups[Title/Abstract] | Study design |
| **#5** | #1 AND #2 AND #3 AND #4 | 157 |

**1.2 Embase**

*Date run: May 2026. Total records retrieved: 286.*

| **Set** | **Search query** | **Records** |
| --- | --- | --- |
| **#1** | 'type 2 diabetes mellitus'/exp OR 'non insulin dependent diabetes mellitus'/exp OR ((('type 2' OR 'type ii' OR t2dm OR niddm OR 'non insulin dependent' OR 'non-insulin-dependent' OR 'adult onset') NEAR/3 (diabet* OR hyperglyc*)):ti,ab,kw) | Population |
| **#2** | 'yoga'/exp OR yog*:ti,ab,kw OR yogasana*:ti,ab,kw OR asana*:ti,ab,kw OR pranayam*:ti,ab,kw OR 'surya namaskar':ti,ab,kw OR suryanamaskar*:ti,ab,kw OR 'hatha yoga':ti,ab,kw OR 'iyengar yoga':ti,ab,kw OR 'integrated yoga':ti,ab,kw OR 'cyclic yoga':ti,ab,kw OR 'yoga therapy':ti,ab,kw OR 'yoga based':ti,ab,kw OR 'yoga-based':ti,ab,kw OR 'yogic practice':ti,ab,kw OR 'yogic practices':ti,ab,kw | Intervention |
| **#3** | 'randomized controlled trial'/exp OR 'controlled clinical trial'/exp OR 'randomization'/exp OR 'single blind procedure'/exp OR 'double blind procedure'/exp OR 'triple blind procedure'/exp OR 'crossover procedure'/exp OR random*:ti,ab,kw OR randomly:ti,ab,kw OR placebo*:ti,ab,kw OR ((single OR double OR triple OR treble) NEAR/3 (blind* OR mask*)):ti,ab,kw OR ((controlled OR control) NEAR/3 (trial* OR study OR studies)):ti,ab,kw OR rct:ti,ab,kw | Study design |
| **#4** | #1 AND #2 AND #3 |  |
| **#5** | #4 NOT ('animal experiment'/exp NOT 'human'/exp) |  |
| **#6** | #5 AND [embase]/lim NOT [medline]/lim | 286 |

**1.3 Web of Science**

*Date run: May 2026. Total records retrieved: 95.*

| **Set** | **Search query** | **Records** |
| --- | --- | --- |
| **Pop.** | "type 2 diabet*" OR "type II diabet*" OR T2DM OR "non-insulin-dependent diabet*" OR NIDDM OR "adult-onset diabet*" | Population |
| **Int.** | yoga OR yogic OR "yoga therapy" OR "yoga intervention" OR "yoga practice" OR "yoga-based" OR yogasana OR asana* OR pranayama OR "yogic breathing" OR "yoga nidra" OR "Sudarshan Kriya" OR "laughter yoga" OR "Hatha yoga" OR "Iyengar yoga" OR Trataka OR "Jyoti-Trataka" | Intervention |
| **Des.** | random* OR trial* OR placebo* OR "controlled trial" OR "clinical trial" OR RCT | Study design |
| **Final** | TS = (Pop. AND Int. AND Des.) | 95 |

**1.4 Cochrane Library**

*Date run: May 2026. Total records retrieved: 225.*

| **Set** | **Search query** | **Records** |
| --- | --- | --- |
| **#1** | MeSH descriptor: [Diabetes Mellitus, Type 2] explode all trees | 27,733 |
| **#2** | ("type 2 diabetes" OR (type NEXT 2 NEXT diabetic*) OR "type II diabetes" OR (type NEXT II NEXT diabetic*) OR T2D OR T2DM OR NIDDM OR "non insulin dependent diabetes" OR "non-insulin-dependent diabetes" OR (non NEXT insulin NEXT dependent NEXT diabetic*) OR "adult onset diabetes" OR "adult-onset diabetes" OR "maturity onset diabetes" OR "maturity-onset diabetes"):ti,ab,kw | 58,789 |
| **#3** | MeSH descriptor: [Yoga] explode all trees | 1,360 |
| **#4** | (yoga OR yogic OR yogasana* OR asana* OR pranayama* OR "yoga therapy" OR (yoga NEXT intervention*) OR "yoga training" OR (yoga NEXT program*) OR (yoga NEXT programme*) OR "yoga based" OR "yoga-based" OR "yogic breathing" OR "laughter yoga" OR "restorative yoga" OR "hatha yoga" OR "iyengar yoga" OR "sudarshan kriya" OR "surya namaskar"):ti,ab,kw | 7,913 |
| **#5** | #1 OR #2 | 62,180 |
| **#6** | #3 OR #4 | 7,913 |
| **#7** | #5 AND #6 | 342 |
| **#8** | (random* OR RCT OR placebo* OR trial* OR "controlled trial" OR "clinical trial" OR "randomized controlled trial" OR "randomised controlled trial"):ti,ab,kw | 1,739,020 |
| **#9** | #7 AND #8 | 225 |

**Supplementary File 2. Characteristics of included studies and background pharmacotherapy**

**Table.2.1 Characteristics of the 28 randomised controlled trials included in the meta-analysis.**

| **Author** | **Year** | **Country** | **Yoga type** | **N (Y/C)** | **Age** | **Female** | **BMI** | **Baseline HbA1c (%)** | **Baseline FBG (mmol/L)** | **Yoga prescription** | **Supervised** | **RoB 2.0** |
| --- | --- | --- | --- | --- | --- | --- | --- | --- | --- | --- | --- | --- |
| **Gordon** | 2008 | Cuba | Hatha yoga | 77/77 | 64±7.4 | 80% | 27.4 | 6.4 | 11.8 | 2.5M×45min×3/wk×24wk | Yes | Some concerns |
| **SkoroKondza** | 2009 | United Kingdom | Community yoga classes | 29/30 | 60±10 | 61% | NR | 7.1 | — | 2.5M×90min×2/wk×12wk | Yes | Some concerns |
| **Pardasany** | 2010 | India | Hatha yoga | 15/15 | NR | 38% | NR | 9.1 | 9.6 | 2.5M×60min×3/wk×12wk | Yes | Some concerns |
| **Vaishali** | 2012 | India | Yoga-based program | 27/30 | 65.8±3.2 | 48% | 27.1 | 10.3 | 9.1 | 2.5M×45min×6/wk×12wk | Yes | Some concerns |
| **Mullur** | 2016 | United States | 10-minute seated yoga | 5/5 | 68.8±6 | 20% | 31.4 | 9.7 | 11.5 | 2M×10min×7/wk×12wk | Yes | High |
| **Gorantla** | 2017 | India | Yoga plus standard treatment (diabetes subgroup) | NR | 37.3±6.2 | 48% | NR | — | 10.8 | 2.5M×45min×3/wk×12wk | NR | Some concerns |
| **Sreedevi** | 2017 | India | Yoga | 32/30 | 52±7.4 | 100% | 24.7 | 9.6 | 9.2 | 2.5M×60min×2/wk×12wk | Yes | Some concerns |
| **Mondal** | 2018 | India | Yogic intervention | NR | 64.7±4 | 100% | 24.3 | — | 9.3 | 2.5M×60min×3/wk×12wk | Yes | Some concerns |
| **Balaji** | 2020 | India | Adjuvant yoga therapy | 34/35 | 51.9±7.6 | 35% | NR | 8.4 | 11.9 | 2.5M×60min×3/wk×16wk | NR | Some concerns |
| **Gupta** | 2020 | India | Yoga-based exercise program | 40/41 | 51.1±8.6 | 48% | 28.8 | 8.5 | 8.9 | 2.5M×60min×1/wk×16wk | Yes | Some concerns |
| **Danasegaran** | 2021 | India | Yoga plus oral antidiabetic drugs | NR | 52±5.2 | 0% | 27.3 | — | 9.3 | 2.5M×40min×5/wk×12wk | NR | Some concerns |
| **Misra** | 2021 | India | Community-based structured yoga | 164/157 | 52.8±10.1 | 60% | NR | 8.8 | 10.8 | 2.5M×50min×2.8/wk×12wk | NR | Some concerns |
| **Ranga** | 2021 | India | Yoga | 50/50 | NR | NR | 24.3 | 8.1 | 8.2 | 2.5M×60min×7/wk×12wk | Yes | Some concerns |
| **Yuniartika** | 2021 | Indonesia | Yoga therapy | NR | NR | 61% | NR | — | 12 | 2.5M×60min×3/wk×12wk | NR | Some concerns |
| **Ismail** | 2022 | Egypt | Jyoti-Trataka | NR | 57.8±3.1 | 57% | 27.6 | — | 9 | 1.3M×55min×7/wk×4wk | Yes | Some concerns |
| **MangalaGowri** | 2022 | India | Integrated yoga therapy | 35/35 | 52.3±10 | NR | 26.9 | 8.6 | 9 | 2.5M×60min×4/wk×17wk | Yes | Some concerns |
| **Vijayashankar** | 2022 | India | Yoga alone | 50/50 | 43.7±6.7 | NR | 31 | 7.5 | 9.2 | 2.5M×60min×7/wk×24wk | Yes | Some concerns |
| **Hirosaki** | 2023 | Japan | Laughter yoga | 21/21 | 71.8±6.4 | 67% | 23.4 | 7.1 | — | 2M×60min×0.7/wk×12wk | Yes | Some concerns |
| **Sharma** | 2023 | India | Yoga intervention | 52/52 | 52.9±7.9 | 38% | 27.8 | 9.3 | 10.1 | 2.5M×40min×5/wk×24wk | Yes | Some concerns |
| **Patil** | 2024 | India | Yogasana and pranayama | NR | NR | 36% | NR | — | 9.9 | 2.5M×30min×7/wk×12wk | Yes | Some concerns |
| **Vidyashree** | 2024 | India | T2DM-specific yoga module | 38/37 | 52.9±8.2 | 18% | 25.7 | 8 | 8.3 | 2.5M×60min×7/wk×12wk | NR | Some concerns |
| **Aski** | 2025 | India | Yoga intervention | 52/52 | 54.9±7.9 | 50% | 27.5 | 7.8 | 8 | 2.5M×60min×5/wk×12wk | Yes | Some concerns |
| **Devi** | 2025 | India | Yoga alone | 30/30 | NR | 100% | NR | 7.7 | 8.6 | 3.3M×60min×5/wk×12wk | Yes | Some concerns |
| **Laimujam** | 2025 | India | Yoga group | 30/30 | 49±7.4 | 60% | NR | 8.2 | 8 | 3.3M×60min×4.2/wk×12wk | Yes | Some concerns |
| **Solmaz** | 2025 | Turkey | Yoga breathing exercise | 35/35 | 49.9±11.7 | 100% | NR | 8.3 | — | 1.5M×60min×2/wk×12wk | Yes | Some concerns |
| **Subramani** | 2025 | India | Yoga intervention | 76/76 | NR | NR | 28.2 | 8.4 | — | 2.5M×35min×0.5/wk×24wk | NR | High |
| **Bayat** | 2026 | Iran | Cyclic yoga | 18/19 | NR | 100% | 31.1 | 8.1 | 9.9 | 3.3M×60min×3/wk×8wk | Yes | Some concerns |
| **Subham** | 2026 | India | Yoga training | 30/30 | 34.1±3.9 | 27% | 22.1 | 8.6 | 5.4 | 2.5M×50min×3/wk×16wk | Yes | Some concerns |

***Note. N (Y/C)*** *= sample size (Yoga / Control);* ***METs*** *refer to the 2024 Adult Compendium of Physical Activities;* ***Yoga prescription*** *is formatted as intensity (MET) × session duration × weekly frequency × programme weeks;* ***NR*** *= not reported.*

**Table 2.2. Background antidiabetic pharmacotherapy and medication-adjustment policies across the 28 included randomized controlled trials.**

Background antidiabetic pharmacotherapy was inconsistently reported across the 28 included trials, and where reported, yoga was delivered as an adjunct to continued background therapy rather than as a replacement. Four trials explicitly required medication to remain unchanged during follow-up (Sharma 2023; Mangala Gowri 2022; Vijayashankar 2022; Laimujam 2025), and two further trials confirmed that no medication change occurred even though no formal no-adjustment rule was pre-specified (Gorantla 2017; Hirosaki 2023). Several trials reported only that participants were on stable agents at entry, without a follow-up policy (Gupta 2020; Aski 2025; Solmaz 2025). Baseline regimens were not reported in three trials (Gordon 2008; Mondal 2018; Subham 2026), and one trial (Yuniartika 2021) contained internally inconsistent statements about whether participants received glucose-lowering medication. Potential medication-related confounding was identified in two trials: Laimujam 2025 showed a marked baseline imbalance in metformin use (40% yoga vs 86.7% control), and Subramani 2025 reported that more yoga participants reduced their oral hypoglycaemic dose than controls (29 vs 15) — because the yoga arm received less pharmacotherapy, this would bias the between-group glycaemic difference toward the null rather than exaggerate the yoga effect. Three insulin-inclusive cohorts were identified (Mullur 2016, an insulin-intensive cohort with 80% chronic kidney disease; Hirosaki 2023; and Vidyashree 2024).

| **Study** | **Country** | **Background glucose-lowering therapy at baseline** | **Medication policy during the intervention period** | **Between-arm comparability of pharmacotherapy** |
| --- | --- | --- | --- | --- |
| Gordon 2008 | Cuba | Not reported (only general diabetes education on diet, exercise and medication was described). | Asymmetric oversight: investigator-managed exercise groups could have treatment adjusted weekly, whereas controls followed their own clinic/physician plan; arm-specific changes not reported. | Not assured — medication oversight differed between arms and arm-specific changes were not reported. |
| Skoro-Kondza 2009 | UK | Non-insulin-treated T2DM; the split between oral agents and diet-only was not reported (insulin-treated patients excluded). | Not reported. | Comparable only in excluding insulin treatment; oral-agent vs diet-only distributions not reported. |
| Pardasany 2010 | India | Oral hypoglycaemic agents in all arms. | OHA continued; no stable-dose or adjustment rule reported. | Broadly comparable at drug-class level; specific agents, doses and changes not reported. |
| Vaishali 2012 | India | Long-term antidiabetic drugs (>15 years); drug class/route not reported. | Both arms continued conventional hypoglycaemic medication; no dose-adjustment rule reported. | Broadly comparable in continued medication; oral vs insulin, doses and changes not reported. |
| Mullur 2016 | USA | Insulin-intensive cohort: basal insulin 100%, prandial insulin 70% (control 3/5, yoga 4/5), metformin 40%, OHA (control 2/5, yoga 1/5); 80% had chronic kidney disease. | Not reported. | No statistically significant baseline medication difference reported, though descriptive differences existed (OHA, prandial insulin); follow-up changes not reported. |
| Gorantla 2017 | India | Standard diabetes treatment, not itemised. | No major medication change occurred during the 12-week study, confirmed by regular contact with subjects. | Broadly comparable under the same standard-treatment framework with no major change; exact drugs/doses not reported. |
| Sreedevi 2017 | India | Pre-existing pharmacological or non-pharmacological treatment; exact drugs/proportions not reported (control usual care included OHA). | OHA could be reduced by 20–50% for any participant with documented hypoglycaemia (random plasma glucose <70 mg/dL, with or without symptoms). | Indeterminate — the reduction rule applied to “any study participant” and no arm-specific numbers were reported; a differential reduction favouring the yoga arm cannot be established. |
| Mondal 2018 | India | Not reported. | Not reported (“continued usual routine activities” describes daily activity, not a medication policy). | Not reported. |
| Balaji 2020 | India | OHA (sulphonylureas / biguanides / glitazones) or insulin. | Both arms continued standard medical management with yoga as an adjunct; no fixed-dose rule reported. | Broadly comparable by protocol; actual drug classes, insulin prevalence, doses and changes by arm not reported. |
| Gupta 2020 | India | Stable oral glucose-lowering agents for 3 months before enrolment (entry criterion). | Not reported — the 3-month stability was an entry criterion, not a follow-up rule. | Comparable on baseline burden (>2 oral agents: 11/41 usual care vs 16/40 yoga, P = 0.209); specific agents/doses/follow-up changes not reported. |
| Danasegaran 2021 | India | Newly diagnosed / treatment-naive at recruitment, then prescribed metformin 500 mg twice/thrice daily ± glimepiride 1 mg/day; other oral drugs and insulin excluded. | Yoga added to the prescribed OAD regimen; control received OAD alone; no unchanged-dose rule stated. | Strong protocol-level comparability (same permitted agents/dose schedules in both arms); actual follow-up changes not reported. |
| Misra 2021 | India | Control arm: medication as prescribed by physicians; yoga arm not reported. | Stated only for the control arm; yoga-arm policy not reported. | Not reported — comparable baseline regimens/policies across arms were not established. |
| Ranga 2021 | India | Oral hypoglycaemic medication (inclusion criterion). | Both arms continued medication; “dose variation if any” was listed as an assessment item but not reported as a policy or result. | Broadly comparable at OHA-exposure level; specific agents/doses/changes not reported. |
| Yuniartika 2021 | Indonesia | Internally inconsistent — eligibility required no insulin or diabetes medication, but the Discussion states the control group continued oral hypoglycaemic medication. | Internally inconsistent (as above). | Cannot be determined; the trial cannot be safely classified as medication-free without author clarification. |
| Ismail 2022 | Egypt | Oral hypoglycaemic drugs in both arms. | Both arms remained on OHA while only the intervention arm received Jyoti-Trataka; no dose-stability/adjustment rule reported. | Broadly comparable in OHA exposure; agents, doses, baseline balance and changes not reported. |
| Mangala Gowri 2022 | India | Diabetic diet plus oral hypoglycaemic agents. | Both arms were expressly advised to maintain their OHA regimen. | Comparable by protocol (both on diabetic diet + OHA with the same maintenance instruction); specific agents/doses not reported. |
| Vijayashankar 2022 | India | Not reported (medication intake/dosage/duration were noted but not presented). | No alterations were made to the ongoing treatment of any group. | Comparable in the no-alteration policy; baseline medication composition/dose balance not reported. |
| Hirosaki 2023 | Japan | Standard diabetes therapy; 3 participants on insulin (1 yoga, 2 control), the remainder on OHA. | All continued pre-study therapy; no change in medical treatment occurred in either group during the study. | Comparable during follow-up (no treatment change); baseline insulin use 1 vs 2; other agent/dose distributions not reported. |
| Sharma 2023 | India | Oral hypoglycaemic agents only (metformin, sulfonylureas, gliptins, others); no insulin. | Participants were explicitly instructed to keep doses constant throughout the study period. | Comparable by explicit protocol (both arms on constant medication); patient-level molecules/doses unavailable. |
| Patil 2024 | India | Medication-treated in both arms (intervention = medicine + yoga + pranayama; control = diet + oral medication). | Both continued background therapy with yoga added to the intervention arm; no stable-dose rule reported. | Broadly comparable in background medication; the control regimen is described inconsistently; agents/doses/changes not reported. |
| Vidyashree 2024 | India | Oral hypoglycaemic drugs with or without insulin (OHD-only 34 yoga / 34 control; insulin + OHD 4 yoga / 3 control). | Medication dosage changes were monitored; no change in the medication score in the yoga group. | Baseline categories closely comparable and counts unchanged; only the yoga arm was explicitly reported unchanged, control-arm dose stability not reported. |
| Aski 2025 | India | Stable oral hypoglycaemic agents at entry (e.g., metformin). | Not reported — no statement that medication remained unchanged during the 12-week intervention. | Comparable only at entry (stable OHA); exact agents/doses/follow-up changes not reported. |
| Devi 2025 | India | Standard care with diabetic medication; exact regimen not reported. | Standard care continued in all groups; no medication-adjustment rule stated. | Broadly comparable at standard-care level; agents/doses/changes not reported. |
| Laimujam 2025 | India | Stable oral antidiabetic therapy for ≥1 year (metformin and other OHA); metformin use 12/30 (40%) yoga vs 26/30 (86.7%) control. | Medication regimens were explicitly not altered; no change in type or dosage was allowed during the study. | Not fully comparable at baseline — no changes were allowed, but metformin use differed markedly (40% yoga vs 86.7% control). |
| Solmaz 2025 | Türkiye | Stable antidiabetic treatment at entry; agents/stability duration not reported (the ≥6 months refers to diabetes duration, not medication stability). | Both arms continued standard pharmacological treatment; no fixed-dose/no-adjustment rule stated. | Broadly comparable in continuation of standard treatment; classes, doses, baseline balance and changes not reported. |
| Subramani 2025 | India | Oral hypoglycaemic agents present; baseline agents/doses/proportions not reported. | No pre-specified adjustment policy, but dose changes were monitored: more intervention participants reduced their OHA dose (n = 29) than controls (n = 15). | Not comparable during follow-up (29 vs 15 reduced OHA); text reports P < 0.05 but Figure 2 reports P < 0.001. More participants in the yoga group reduced their OHA dose than in the control group. This difference could attenuate the between-group glycemic contrast, although the dose reductions may also have followed clinical improvement. |
| Bayat 2026 | Iran | Metformin in all participants (1–3 tablets daily); insulin-dependent patients excluded. | Not reported — medication was reviewed before baseline but no follow-up adjustment rule was stated. | Comparable in the metformin class; dose distribution/follow-up changes not reported. |
| Subham 2026 | India | Not reported (HbA1c <9% was an eligibility criterion and does not establish medication use). | Not reported. | Not reported. |

*Abbreviations: NR, not reported; OAD, oral antidiabetic drug; OHA, oral hypoglycaemic agent; OHD, oral hypoglycaemic drug; T2DM, type 2 diabetes mellitus. Information was extracted from the full texts of the included trials; “not reported” denotes that the corresponding detail was not stated in the published article. Where a statement could not be unambiguously attributed to one arm or to the follow-up period, the entry is marked indeterminate or inconsistent rather than inferred.*

**Supplementary File 3. Risk of Bias 2.0 assessment (by outcome)**

**3.1 RoB 2.0 — HbA1c (22 studies)**

**Table. Risk of Bias 2.0 judgments for studies contributing to the HbA1c meta-analysis.**

| **Study** | **D1** | **D2** | **D3** | **D4** | **D5** | **Overall** |
| --- | --- | --- | --- | --- | --- | --- |
| **Gordon et al. 2008** | Some | Some | Some | Low | Some | Some |
| **SkoroKondza et al. 2009** | Some | Some | Some | Low | Some | Some |
| **Pardasany et al. 2010** | Some | Some | Some | Low | Some | Some |
| **Vaishali et al. 2012** | Some | Some | Some | Low | Some | Some |
| **Mullur et al. 2016** | Some | Some | High | Low | Some | High |
| **Sreedevi et al. 2017** | Some | Some | Some | Low | Some | Some |
| **Balaji et al. 2020** | Some | Some | Some | Low | Some | Some |
| **Gupta et al. 2020** | Some | Some | Some | Low | Some | Some |
| **Misra et al. 2021** | Some | Some | Some | Low | Some | Some |
| **Ranga et al. 2021** | Some | Some | Some | Low | Some | Some |
| **MangalaGowri et al. 2022** | Some | Some | Some | Low | Some | Some |
| **Vijayashankar et al. 2022** | Some | Some | Some | Low | Some | Some |
| **Hirosaki et al. 2023** | Some | Some | Some | Low | Some | Some |
| **Sharma et al. 2023** | Some | Some | Some | Low | Some | Some |
| **Vidyashree et al. 2024** | Some | Some | Some | Low | Some | Some |
| **Aski et al. 2025** | Some | Some | Some | Low | Some | Some |
| **Devi et al. 2025** | Some | Some | Some | Low | Some | Some |
| **Laimujam et al. 2025** | Some | Some | Some | Low | Some | Some |
| **Solmaz et al. 2025** | Some | Some | Some | Low | Some | Some |
| **Subramani et al. 2025** | Some | Some | High | Low | Some | High |
| **Bayat et al. 2026** | Some | Some | Some | Low | Some | Some |
| **Subham et al. 2026** | Some | Some | Some | Low | Some | Some |

***Note. D1*** *= randomisation process;* ***D2*** *= deviations from intended interventions;* ***D3*** *= missing outcome data;* ***D4*** *= measurement of the outcome;* ***D5*** *= selection of the reported result.* ***Some*** *= "Some concerns";* ***Low*** *= "Low risk of bias";* ***High*** *= "High risk of bias".*

**3.2 RoB 2.0 — Fasting blood glucose (24 studies)**

**Table. Risk of Bias 2.0 judgments for studies contributing to the fasting blood glucose meta-analysis.**

| **Study** | **D1** | **D2** | **D3** | **D4** | **D5** | **Overall** |
| --- | --- | --- | --- | --- | --- | --- |
| **Gordon et al. 2008** | Some | Some | Some | Low | Some | Some |
| **Pardasany et al. 2010** | Some | Some | Some | Low | Some | Some |
| **Vaishali et al. 2012** | Some | Some | Some | Low | Some | Some |
| **Mullur et al. 2016** | Some | Some | High | Low | Some | High |
| **Gorantla et al. 2017** | Some | Some | Some | Low | Some | Some |
| **Sreedevi et al. 2017** | Some | Some | Some | Low | Some | Some |
| **Mondal et al. 2018** | Some | Some | Some | Low | Some | Some |
| **Balaji et al. 2020** | Some | Some | Some | Low | Some | Some |
| **Gupta et al. 2020** | Some | Some | Some | Low | Some | Some |
| **Danasegaran et al. 2021** | Some | Some | Some | Low | Some | Some |
| **Misra et al. 2021** | Some | Some | Some | Low | Some | Some |
| **Ranga et al. 2021** | Some | Some | Some | Low | Some | Some |
| **Yuniartika et al. 2021** | Some | Some | Some | Low | Some | Some |
| **Ismail et al. 2022** | Some | Some | Some | Low | Some | Some |
| **MangalaGowri et al. 2022** | Some | Some | Some | Low | Some | Some |
| **Vijayashankar et al. 2022** | Some | Some | Some | Low | Some | Some |
| **Sharma et al. 2023** | Some | Some | Some | Low | Some | Some |
| **Patil et al. 2024** | Some | Some | Some | Low | Some | Some |
| **Vidyashree et al. 2024** | Some | Some | Some | Low | Some | Some |
| **Aski et al. 2025** | Some | Some | Some | Low | Some | Some |
| **Devi et al. 2025** | Some | Some | Some | Low | Some | Some |
| **Laimujam et al. 2025** | Some | Some | Some | Low | Some | Some |
| **Bayat et al. 2026** | Some | Some | Some | Low | Some | Some |
| **Subham et al. 2026** | Some | Some | Some | Low | Some | Some |

***Note. D1*** *= randomisation process;* ***D2*** *= deviations from intended interventions;* ***D3*** *= missing outcome data;* ***D4*** *= measurement of the outcome;* ***D5*** *= selection of the reported result.* ***Some*** *= "Some concerns";* ***Low*** *= "Low risk of bias";* ***High*** *= "High risk of bias".*

**Supplementary File 4. Risk of Bias 2.0 summary bar plots (by outcome)**

The figures below summarise the distribution of risk-of-bias judgments across the five domains and the overall rating, presented separately for each outcome. Each bar shows the proportion of studies rated as "Low" (green), "Some concerns" (yellow), or "High risk" (red) within that subset.

**4.1 Bar plot — HbA1c subset**

**Figure. Risk of Bias 2.0 summary for the HbA1c meta-analysis (22 studies).**

**4.2 Bar plot — Fasting blood glucose subset**

**Figure. Risk of Bias 2.0 summary for the fasting blood glucose meta-analysis (24 studies).**

**Supplementary File 5. Posterior distributions of pooled effects and heterogeneity**

**Figure .** Posterior distributions for HbA1c: (a) pooled mean difference of yoga versus control; (b) heterogeneity standard deviations at the study level and effect-size level.

**Figure .** Posterior distributions for fasting blood glucose: (a) pooled mean difference of yoga versus control; (b) heterogeneity standard deviations at the study level and effect-size level.

**Supplementary File 6. Posterior predictive checks**

**Figure. Posterior predictive check for HbA1c: observed mean differences (dark line) overlaid on 100 replicated datasets simulated from the fitted posterior (light lines).**

**Figure. Posterior predictive check for fasting blood glucose: observed mean differences (dark line) overlaid on 100 replicated datasets simulated from the fitted posterior (light lines).**

**Supplementary File 7. Moderator analyses**

Subgroup comparisons and meta-regression of treatment effect on study-level moderators. Part A presents subgroup analyses for three categorical moderators that passed the balance criteria (country, supervision, and WHO weekly-dose threshold). Part B presents meta-regression of seven continuous moderators, with linear and spline (k = 3, k = 4) specifications compared via leave-one-out information criterion (LOO-IC).

***A. Subgroup analyses***

| **Outcome** | **Moderator** | **Subgroup** | **k** | **MD [95% CrI]** | **pd** | **Interaction BF** |
| --- | --- | --- | --- | --- | --- | --- |
| **HbA1c (%)** | Country (India vs Non-India) | India | 16 | −0.61 [−0.89, −0.34] | 1.000 | 0.30 |
|  |  | Non-India | 6 | −0.66 [−1.14, −0.23] | 0.996 |  |
|  | WHO dose threshold | < 600 MET-min/wk | 14 | −0.63 [−0.96, −0.31] | 1.000 | 0.26 |
|  | (≥ 600 vs < 600) | ≥ 600 MET-min/wk | 8 | −0.63 [−0.91, −0.35] | 1.000 |  |
| **FBG (mmol/L)** | Country (India vs Non-India) | India | 19 | −1.33 [−1.74, −0.94] | 1.000 | 0.29 |
|  |  | Non-India | 5 | −1.46 [−2.91, −0.06] | 0.979 |  |
|  | Supervised vs supervised + home | Supervised only | 18 | −1.36 [−1.86, −0.88] | 1.000 | 0.19 |
|  |  | Supervised + home | 6 | −1.36 [−2.13, −0.62] | 0.999 |  |
|  | WHO dose threshold | < 600 MET-min/wk | 16 | −1.25 [−1.77, −0.75] | 1.000 | 0.23 |
|  | (≥ 600 vs < 600) | ≥ 600 MET-min/wk | 8 | −1.50 [−2.16, −0.82] | 1.000 |  |

***B. Meta-regression (standardised β per 1 SD change)***

| **Outcome** | **Moderator** | **k** | **Unique values** | **Best model** | **β [95% CrI]** | **pd** | **ΔLOOIC (best − lin)** |
| --- | --- | --- | --- | --- | --- | --- | --- |
| **HbA1c (%)** | Baseline HbA1c (%) | 22 | 22 | k3 | **−0.20 [−0.39, −0.02]*** | 0.984 | −2.32 |
|  | Frequency (/wk) | 22 | 11 | linear | −0.12 [−0.31, +0.08] | 0.882 | — |
|  | Session duration (min) | 22 | 7 | linear | +0.15 [−0.09, +0.39] | 0.889 | — |
|  | Duration (weeks) | 22 | 5 | linear | −0.01 [−0.20, +0.18] | 0.528 | — |
|  | Mean age (y) | 17 | 16 | linear | +0.02 [−0.23, +0.27] | 0.565 | — |
|  | Female % | 18 | 15 | linear | +0.08 [−0.15, +0.31] | 0.771 | — |
|  | BMI (kg/m²) | 15 | 15 | linear | −0.08 [−0.35, +0.20] | 0.722 | — |
| **FBG (mmol/L)** | Baseline FBG (mmol/L) | 24 | 24 | k3 | **−0.49 [−0.85, −0.15]*** | 0.996 | −5.94 |
|  | Frequency (/wk) | 24 | 9 | k3 | −0.19 [−0.60, +0.20] | 0.826 | −0.06 |
|  | Session duration (min) | 24 | 7 | k3 | **+0.95 [+0.54, +1.35]*** | 1.000 | −0.46 |
|  | Duration (weeks) | 24 | 6 | linear | −0.22 [−0.61, +0.15] | 0.878 | — |
|  | Mean age (y) | 18 | 17 | linear | −0.35 [−0.87, +0.15] | 0.919 | — |
|  | Female % | 21 | 18 | linear | +0.35 [−0.03, +0.75] | 0.965 | — |
|  | BMI (kg/m²) | 16 | 16 | k3 | −0.47 [−1.04, +0.08] | 0.953 | −2.22 |

***Note. Effect size****: mean difference (MD) on the original scale; negative values favour yoga.* ***** *indicates 95% credible interval excludes zero.* ***Interaction BF****: Bayes factor for the model with treatment × moderator interaction over the additive model (BF₁₀ < 1 indicates no support for moderation).* ***Best model*** *selected by minimum LOO-IC among linear, spline k = 3, and spline k = 4 (when ≥ 6 unique values).* ***ΔLOOIC****: difference between the best model and the linear model (negative values favour the best non-linear model).* ***WHO dose*** *threshold: 600 MET-minutes per week. The session-duration analysis was exploratory and based on seven unique duration values. Because the models used aggregate study-level data and did not simultaneously adjust for study size, risk of bias, adherence, or correlated intervention characteristics, the association should not be interpreted as causal or as establishing an optimal session duration.*

C. ***Robustness of the FBG session-duration meta-regression coefficient.***

| **Set** | ***k*** | ***β* (session)** | **95% CrI** | ***pd*** |
| --- | --- | --- | --- | --- |
| Full sample | 24 | 0.95 | 0.52 to 1.36 | 100% |
| Excluding shortest-session study (Mullur 2016, *n* = 10) | 23 | 0.67 | 0.34 to 1.01 | 100% |
| Excluding below-median-sized studies | 12 | 0.74 | 0.30 to 1.16 | 99.9% |
| Weighted by study precision (1/SE) | 24 | 0.97 | 0.58 to 1.36 | 100% |

*Note.* *β* is the standardized coefficient for session duration in the FBG meta-regression. Positive values indicate that longer sessions are associated with smaller reductions, so an attenuated *β* corresponds to a weaker “shorter is better” pattern. The coefficient stayed above zero across all checks. CrI, credible interval. *pd*, probability of direction.

**Supplementary File 8. Funnel plots**

**Figure. Contour-enhanced funnel plots for the two glycaemic outcomes: (a) HbA1c; (b) fasting blood glucose. Inner band: pseudo-95% confidence interval; outer band: pseudo-99% confidence interval. The dashed horizontal line indicates the pooled estimate.**

**Supplementary File 9. Egger and Begg publication-bias tests**

**Table. Statistical tests for funnel-plot asymmetry.**

| **Outcome** | **k** | **Pooled MD** | **Egger t** | **df** | **Egger p** | **Begg τ** | **Begg p** |
| --- | --- | --- | --- | --- | --- | --- | --- |
| **HbA1c (%)** | 22 | −0.642 | −1.178 | 20 | 0.253 | −0.203 | 0.197 |
| **FBG (mmol/L)** | 24 | −1.381 | −3.902 | 22 | **<0.001** | +0.014 | 0.941 |

***Note. Effect size*** *is the study-level mean difference yᵢ = MD_yoga − MD_control, with SE(yᵢ) = √(SE_yoga² + SE_control²).* ***Egger test*** *is a regression of standardised effect on precision; p < 0.05 indicates funnel-plot asymmetry.* ***Begg test*** *is a Kendall rank correlation between effect size and standard error.* ***Interpretation:*** *For HbA1c, neither test reached significance, supporting funnel symmetry. For FBG, Egger's test was highly significant (p < 0.001) while Begg's was not (p = 0.941), suggesting some asymmetry that may reflect small-study effects, true heterogeneity, or differential methodological quality in smaller trials. This finding contributed to the GRADE downgrade for publication bias.*

**Supplementary File 10. Leave-one-out sensitivity analysis (numerical results)**

The three-level Bayesian meta-analysis was re-fitted after excluding each study in turn. Pooled mean difference (MD) and 95% credible interval (CrI) are shown. The "Full" row reproduces the primary analysis.

*A. HbA1c (%). Largest positive deviation: +0.061 (Sharma 2023); largest negative deviation: −0.036 (Subramani 2025).*

| **Excluded study** | **Estimate** | **Est. error** | **95% CrI lower** | **95% CrI upper** |
| --- | --- | --- | --- | --- |
| ***None (Full)*** | ***−0.636*** | ***0.116*** | ***−0.870*** | ***−0.415*** |
| Gordon 2008 | −0.615 | 0.117 | −0.852 | −0.389 |
| Sreedevi 2017 | −0.644 | 0.119 | −0.886 | −0.410 |
| Vidyashree 2024 | −0.647 | 0.123 | −0.894 | −0.408 |
| SkoroKondza 2009 | −0.659 | 0.119 | −0.893 | −0.426 |
| Subham 2026 | −0.645 | 0.124 | −0.889 | −0.399 |
| Pardasany 2010 | −0.662 | 0.120 | −0.910 | −0.429 |
| Vijayashankar 2022 | −0.612 | 0.117 | −0.851 | −0.385 |
| Gupta 2020 | −0.653 | 0.121 | −0.899 | −0.418 |
| Vaishali 2012 | −0.628 | 0.119 | −0.868 | −0.398 |
| Devi 2025 | −0.631 | 0.122 | −0.875 | −0.397 |
| MangalaGowri 2022 | −0.610 | 0.114 | −0.840 | −0.386 |
| Sharma 2023 | −0.570 | 0.102 | −0.777 | −0.369 |
| Hirosaki 2023 | −0.652 | 0.120 | −0.889 | −0.416 |
| Balaji 2020 | −0.590 | 0.116 | −0.828 | −0.368 |
| Ranga 2021 | −0.666 | 0.120 | −0.907 | −0.435 |
| Laimujam 2025 | −0.640 | 0.120 | −0.880 | −0.412 |
| Subramani 2025 | −0.674 | 0.115 | −0.904 | −0.446 |
| Solmaz 2025 | −0.609 | 0.118 | −0.850 | −0.380 |
| Aski 2025 | −0.638 | 0.125 | −0.894 | −0.396 |
| Misra 2021 | −0.648 | 0.118 | −0.886 | −0.422 |
| Mullur 2016 | −0.634 | 0.117 | −0.870 | −0.407 |
| Bayat 2026 | −0.618 | 0.116 | −0.852 | −0.383 |

*B. FBG (mmol/L). Largest positive deviation: +0.061 (Vijayashankar 2022); largest negative deviation: −0.070 (Subham 2026).*

| **Excluded study** | **Estimate** | **Est. error** | **95% CrI lower** | **95% CrI upper** |
| --- | --- | --- | --- | --- |
| ***None (Full)*** | ***−1.356*** | ***0.190*** | ***−1.743*** | ***−0.999*** |
| Gordon 2008 | −1.311 | 0.190 | −1.692 | −0.946 |
| Sreedevi 2017 | −1.394 | 0.194 | −1.782 | −1.015 |
| Vidyashree 2024 | −1.407 | 0.192 | −1.788 | −1.037 |
| Subham 2026 | −1.429 | 0.188 | −1.813 | −1.071 |
| Pardasany 2010 | −1.374 | 0.200 | −1.779 | −0.986 |
| Gorantla 2017 | −1.367 | 0.212 | −1.802 | −0.966 |
| Ismail 2022 | −1.420 | 0.194 | −1.812 | −1.054 |
| Vijayashankar 2022 | −1.298 | 0.190 | −1.688 | −0.929 |
| Gupta 2020 | −1.397 | 0.197 | −1.788 | −1.017 |
| Danasegaran 2021 | −1.337 | 0.200 | −1.751 | −0.948 |
| Yuniartika 2021 | −1.347 | 0.196 | −1.745 | −0.971 |
| Vaishali 2012 | −1.319 | 0.199 | −1.745 | −0.938 |
| Devi 2025 | −1.371 | 0.206 | −1.795 | −0.975 |
| MangalaGowri 2022 | −1.316 | 0.188 | −1.693 | −0.950 |
| Sharma 2023 | −1.348 | 0.202 | −1.760 | −0.951 |
| Balaji 2020 | −1.305 | 0.188 | −1.686 | −0.944 |
| Ranga 2021 | −1.404 | 0.205 | −1.822 | −1.018 |
| Laimujam 2025 | −1.320 | 0.201 | −1.732 | −0.936 |
| Aski 2025 | −1.370 | 0.210 | −1.799 | −0.966 |
| Patil 2024 | −1.325 | 0.192 | −1.717 | −0.958 |
| Misra 2021 | −1.378 | 0.202 | −1.794 | −0.988 |
| Mullur 2016 | −1.344 | 0.193 | −1.737 | −0.975 |
| Mondal 2018 | −1.403 | 0.200 | −1.801 | −1.025 |
| Bayat 2026 | −1.353 | 0.196 | −1.749 | −0.967 |

***Note.*** *No single study exclusion produced a pooled estimate outside the 95% credible interval of the primary analysis, confirming robustness of the meta-analytic conclusion.*

**Supplementary File 11. Leave-one-out sensitivity (visualisation)**

**Figure. Leave-one-out sensitivity for (a) HbA1c and (b) fasting blood glucose. Each row shows the pooled mean difference and 95% credible interval when that study is excluded. The vertical orange line indicates the primary pooled estimate.**

**Supplementary File 12. Pre-post correlation sensitivity (numerical results)**

The primary analysis assumed a pre-post correlation r = 0.5 (Follmann et al., 1992). Re-running the meta-analysis with r = 0.3, 0.7, and 0.9 quantifies the dependency of the pooled estimate on this assumption.

*A. HbA1c (%). Range of pooled estimates across r values: 0.07%.*

| **Assumed r** | **Estimate** | **Est. error** | **95% CrI lower** | **95% CrI upper** |
| --- | --- | --- | --- | --- |
| 0.3 | −0.605 | 0.110 | −0.836 | −0.399 |
| ***0.5 (primary)*** | ***−0.627*** | ***0.115*** | ***−0.869*** | ***−0.420*** |
| 0.7 | −0.652 | 0.118 | −0.881 | −0.420 |
| 0.9 | −0.677 | 0.127 | −0.926 | −0.429 |

*B. FBG (mmol/L). Range of pooled estimates across r values: 0.13 mmol/L.*

| **Assumed r** | **Estimate** | **Est. error** | **95% CrI lower** | **95% CrI upper** |
| --- | --- | --- | --- | --- |
| 0.3 | −1.342 | 0.194 | −1.717 | −0.960 |
| ***0.5 (primary)*** | ***−1.374*** | ***0.197*** | ***−1.773*** | ***−0.986*** |
| 0.7 | −1.416 | 0.195 | −1.789 | −1.021 |
| 0.9 | −1.472 | 0.196 | −1.834 | −1.054 |

***Note.*** *The total range of pooled estimates across r = 0.3 to 0.9 was 0.072% for HbA1c and 0.131 mmol/L for FBG, well within the credible interval of the primary analysis. All 95% CrIs remained entirely below zero, confirming insensitivity of the qualitative conclusion to the correlation assumption.*

**Supplementary File 13. Pre-post correlation sensitivity (visualisation)**

**Figure. Pooled mean difference and 95% credible interval across four assumed pre-post correlations (r = 0.3, 0.5, 0.7, 0.9) for (a) HbA1c and (b) fasting blood glucose.**

**Supplementary File 14. Sensitivity analysis excluding studies at high overall risk of bias**

The primary three level Bayesian model was refitted after excluding studies rated at high overall risk of bias for each outcome. Mullur 2016 and Subramani 2025 were excluded from the HbA1c analysis. Mullur 2016 was excluded from the FBG analysis.

**Three-level Bayesian sensitivity analysis excluding studies rated at high overall risk of bias (RoB 2.0).**

| **Analysis set** | **k** | **MD** | **95% CrI** | **95% PI** | **pd** | **τ(study)** |
| --- | --- | --- | --- | --- | --- | --- |
| HbA1c — all studies | 22 | −0.634 | −0.870, −0.414 | −1.163, −0.101 | 100% | 0.217 |
| HbA1c — excl. high-risk | 20 | −0.673 | −0.911, −0.454 | −1.252, −0.098 | 100% | 0.243 |
| FBG — all studies | 24 | −1.358 | −1.733, −0.986 | −2.726, −0.035 | 100% | 0.612 |
| FBG — excl. high-risk | 23 | −1.341 | −1.732, −0.973 | −2.658, −0.081 | 100% | 0.590 |

*High-risk studies were Mullur 2016 and Subramani 2025 for HbA1c, and Mullur 2016 for FBG. Units: HbA1c, %; FBG, mmol/L. MD, mean difference; CrI, credible interval; PI, prediction interval; pd, probability of direction; τ(study), between-study standard deviation. Model: md | se(se_md) ~ 1 + group + (1 | studyID/es_id).*

*Supplementary Figure A. Pooled mean difference (95% CrI) for the primary (all-studies) and high-risk-excluded analyses, by outcome.*

**Supplementary File 15. Publication-bias adjustment (trim-and-fill and PET-PEESE)**

Secondary publication bias adjustment analyses were conducted for both glycemic outcomes. The trim and fill method used the L0 and R0 estimators. PET PEESE used PEESE when the PET intercept was significant and PET otherwise. The PET intercept was significant for FBG (p = 0.010) but not for HbA1c (p = 0.140).

**Publication-bias adjustment of the pooled effect using trim-and-fill (L0, R0) and PET-PEESE.**

| **Outcome** | **Method** | **Imputed k** | **Adjusted MD** | **95% CI** |
| --- | --- | --- | --- | --- |
| HbA1c | Observed (REML) | — | −0.642 | −0.854, −0.431 |
| HbA1c | Trim-and-fill (L0) | 3 | −0.536 | −0.777, −0.294 |
| HbA1c | Trim-and-fill (R0) | 0 | −0.642 | −0.854, −0.431 |
| HbA1c | PET-PEESE | — | −0.315 | −0.733, 0.103 |
| FBG | Observed (REML) | — | −1.381 | −1.731, −1.031 |
| FBG | Trim-and-fill (L0) | 4 | −1.240 | −1.595, −0.885 |
| FBG | Trim-and-fill (R0) | 5 | −1.172 | −1.538, −0.805 |
| FBG | PET-PEESE | — | −1.273 | −1.656, −0.889 |

*Study-level effect sizes were calculated as the between-arm difference in pre-to-post change (yoga minus control). Negative values favor yoga. The PET PEESE conditional estimate used PEESE when the PET intercept was significant and PET otherwise. The PET intercept was significant for FBG (p = 0.010) but not for HbA1c (p = 0.140). Units are percentage points for HbA1c and mmol/L for FBG. CI means confidence interval.*

*Figure 1. Trim-and-fill funnel plot for FBG (open points are imputed studies).*

*Figure 2. Trim-and-fill funnel plot for HbA1c.*

**Supplementary File 16. GRADE evidence profile.**

| **Domain** | **HbA1c (22 studies, 44 arms)** | **FBG (24 studies, 48 arms)** |
| --- | --- | --- |
| Study design (starting certainty) | Randomized trials (High) | Randomized trials (High) |
| Risk of bias | Serious (−1) | Serious (−1) |
| Inconsistency | Not serious | Not serious |
| Indirectness | Not serious | Not serious |
| Imprecision | Not serious | Not serious |
| Publication bias | Serious (−1) | Serious (−1) |
| Pooled MD [95% CrI] | −0.64 [−0.87, −0.41] | −1.36 [−1.75, −1.00] |
| Prediction interval | −1.17 to −0.09 | −2.72 to −0.03 |
| Certainty | ⊕⊕○○ Low | ⊕⊕○○ Low |

**Per-domain rationale.**

Per-domain rationale.

1. Risk of bias (both, serious). No study reached low overall risk, with some concerns in 20 of 22 HbA1c and 23 of 24 FBG studies and high risk in 2 and 1, driven mainly by the unavoidable absence of blinding and by incomplete reporting. Outcome measurement was at low risk in every study because HbA1c and FBG are objective standardized measures. Excluding high-risk studies left the pooled estimates essentially unchanged (HbA1c −0.67 vs −0.64, FBG −1.34 vs −1.36). A two-level downgrade was rejected because only 2 of 22 and 1 of 24 studies were high risk, their removal did not alter the estimate, and no single domain reached high risk across the majority of studies.
2. Inconsistency (both, not serious). All estimates favoured yoga (pd = 100%) and both prediction intervals remained below the null. HbA1c heterogeneity was low to moderate (τ_study = 0.21). FBG heterogeneity was higher (τ_study = 0.62) with a wide prediction interval, but part of it was explained by baseline FBG as an identified moderator (β = −0.49) and the direction of effect was uniform.
3. Indirectness (both, not serious). The population, intervention, comparator, and objective outcomes matched the review question directly, so no downgrade was applied for indirectness. Two prose-level limitations were considered under this domain. First, 20 of 28 trials were conducted in India. Second, the country, supervision, and weekly-dose subgroup comparisons each returned an interaction BF₁₀ below 1 but rested on small strata, so these results indicate an absence of evidence for effect modification rather than evidence of equivalence. Because the studied population matched the review question and the mechanisms by which yoga affects glycaemia are not population-specific, both were treated as applicability limitations, reported in Section 4.4, rather than as PICO indirectness.
4. Imprecision (both, not serious). Both credible intervals excluded the null with pd = 100%. For HbA1c the point estimate exceeded the 0.5% threshold. For FBG the least-benefit bound coincided with the 1.0 mmol/L threshold.
5. Publication bias (both, serious). For HbA1c, Egger's (p = 0.253) and Begg's (p = 0.197) tests were non-significant and trim-and-fill estimates stayed below zero (−0.54 and −0.64), but the PET-PEESE conditional estimate was attenuated and crossed the null (−0.32, −0.73 to 0.10), so small-study effects could not be excluded and one level was deducted. For FBG, Egger's test indicated asymmetry (p < 0.001) and trim-and-fill and PET-PEESE both attenuated the effect (−1.24 to −1.27), consistent with small-study effects.

CrI, credible interval. pd, probability of direction. τ_study, between-study heterogeneity standard deviation. MD, mean difference (HbA1c %, FBG mmol/L, negative favours yoga).
